# A methodologically sound survey of Chinese consumers’ willingness to participate in courier, express, and parcel companies’ green logistics

**DOI:** 10.1371/journal.pone.0255532

**Published:** 2021-07-30

**Authors:** Muhammad Jawad Sajid, Ernesto D. R. Santibanez Gonzalez, Jie Zhan, Xiaohong Song, Yubo Sun, Jing Xie

**Affiliations:** 1 School of Engineering Management, Xuzhou University of Technology, Xuzhou, Jiangsu, China; 2 Department of Industrial Engineering, Universidad de Talca, Curicó, Chile; 3 Anhui Vocational College of Electronics and Information Technology, Bengbu, Anhui, China; Shenzhen University, CHINA

## Abstract

The environmental footprint of courier, express, and parcel (CEP) logistics is significant and growing, owing to increased e-commerce. Consumer willingness to participate in the green logistics of CEPs, however, has been understudied. This study addresses this knowledge gap by surveying 155 Chinese consumers about their willingness to participate in CEP green logistics. Additionally, this research identifies some technical issues with previous survey research. Three main factors were extracted after the data were tested for reliability and validity using exploratory factor analysis with principal axis factor extraction and confirmatory factor analysis with diagonally weighted least squares. Consumer willingness is positively correlated with economic (8 items), operational (3 items), and social (3 items) factors, with a statistical significance of p < 0.001. Of all the factors, the strongest correlation, 0.67 (95% CI = 0.57, 0.75; p < 0.001; N = 155), exists between economic factors and consumer willingness. The results of a multinomial logistic regression analysis suggest that all consumers are highly unlikely to participate in economic factors, while they are highly likely to positively commit to operational and social factors. Therefore, it is recommended that the government provides monetary incentives to CEP companies to adopt green logistics, such as tax reductions and subsidies, to reduce the costs of green logistics. Meanwhile, the CEP industry could provide some direct and indirect incentives to consumers to re-use, recycle, and share materials, and to spend time learning about express enterprises’ green logistics, to increase consumer participation in economic factors.

## 1. Introduction

Logistics operations have a significant impact on greenhouse gas emissions [1]. Supply chain operations, particularly logistic activities, compared to other activities, release large amounts of toxic gases and waste into the atmosphere, threatening both the health of the planet and human life [2]. It is universally known that logistics is the backbone of the courier, express, and parcel (CEP) industry, an industry with a major impact on the carbon footprint of a country [1]. While production and consumption-side strategies may be used to address the carbon footprint [3], the former strategies do not consider the environmental impact of consumer choice, use, discard, and growth [4]. Green logistics aligns the logistical roles of transport, storage, and packaging with the environmental objectives of mitigating resource use and pollution [1, 5]. The green consumption idea has emerged as a central element in academic and policy discussions on the environmental impact of consumption [6]. Consumers play a key role in the development of green supply chains [7], while modern culture is based on consumer eco-friendly consumption [8]. Therefore, without developing a deep understanding of the relevant factors in consumers’ willingness to participate in the CEP industry’s green logistics, the industry’s competitive integration of green logistics may not be possible.

In 2019, China’s parcel shipping volume amounted to 63.5 billion parcels, accounting for approximately 62% of the total global parcel shipping volume [9]. The Chinese CEP industry has experienced an average 35% increase in the number of parcels in recent years [10]. Driven by growth in e-commerce, the CEP industry in China is estimated to have exceeded 8% ‘compound annual growth rate (CAGR)’ [11]. According to China’s postal service regulator, during 2020, the Chinese CEP industry handled a total of 74 billion packages, an average of 52 packages per person [10]. All of these indicators predict an ever-increasing environmental impact of China’s CEP industry. The 2019 China Express Green Development Status and Trend Report, published at the 2019 China Express Last Mile Summit, shows that China’s annual volume of express delivery business has increased, the absolute amount of packaging use continues to grow, resource consumption and pollution problems are looming, and that the pressure on environmental protection is increasing [12]. At this summit, Liu Jun, Deputy Director of the State Post Office, said that the CEP industry was working hard to solve the environmental problems of express packages, and that it had achieved initial results in reducing the size of express packages and recycling materials [13]. Consumer willingness to participate in green logistics is key to the development of green logistics in the CEP industry; consumers can promote green logistics in the CEP industry through their own individual and societal actions, and as a pressure factor for the operational efficiency of the CEP industry’s logistics.

However, in practice, Chinese consumer participation in green logistics is relatively low, while the green product market is not competitive and is confined to a few niche consumers [14]. Therefore, to promote it, it is necessary to analyse and address the consumers’ specific reasons for not actively participating in the CEP industry’s green logistics. This understanding can, in turn, help improve pricing, taxation, and other incentive policies [14] to increase consumer willingness to engage in green logistics in China’s CEP industry. Besides government policy making, the role of the CEP industry in prompting consumer green logistics awareness and willingness (or positive attitude) cannot be ignored because, ultimately, the cost of green logistics is either paid by the industry or is transferred to the final consumers in terms of higher prices. Consequently, it is critical for the CEP industry to first understand the key socioeconomic factors that directly or indirectly impact consumer willingness towards green logistics, before concerted efforts are made to promote consumer green behaviour and overcome the consumer concerns or hurdles that negatively impact consumer willingness to participate in the CEP industry’s green logistics.

There is, however, scant literature on consumer willingness to participate in CEP firms’ green logistics, especially in China. Additionally, there are technical issues with some survey studies on related topics that have been published in high-impact journals, which are addressed in this study. A deep understanding of the different items that affect Chinese consumers’ willingness to participate in CEP green logistics could help the government in its development of the CEP industry’s green logistics policy, which would help achieve China’s green development goals. Meanwhile, such knowledge would benefit CEP industries in their promotion of consumer willingness to participate in green logistics (e.g. through monetary incentives, awareness, and environmental consciousness), which, in turn, would ensure positive consumer reaction (response), thus reducing the risk of any adverse effects on consumer demand/satisfaction (for example, consumers might not agree to pay extra or change to green consumption behaviour). This study could, in addition, help define policies for the financial and moral benefit of CEP consumers (from monetary incentives etc., and social obligations, respectively). Therefore, this study has the potential to guide the development of a multi-stake holder, win-win strategy for all the key stakeholders. Multi-stakeholder oriented environmental management and policies are essential for the realisation of environmental and social gains [15], all of which issues are discussed in detail in this study. Table 1 presents a comprehensive summary of the literature on the participation of consumers, employees, and firms in green logistics.

**Table 1:** Summary of the literature on the participation of consumers, employees, and firms in green logistics.

Category	Reference	Year	Study
Coal logistics			
	[16]	2019	Survey on Chinese large and medium sized companies’ willingness to implement green coal logistics
Packaging			
	[17]	2019	Survey on Chinese consumer willingness to pay for green packaging from logistics industry.
Reverse logistics			
	[18]	2016	Survey on European consumer behaviour (willingness to pay) towards reverse green supply chain.
	[19]	2016	Survey on consumer intention to return e-waste in context of reverse green logistics.
Food industry			
	[20]	2021	Survey on Indian young consumer green values impact on green behaviour towards green food logistics.
	[21]	2016	Survey on the adoption of in food processing industries in Malaysia.
Manufacturing industry			
	[22]	2012	Survey on adoption of green logistics management by Chinese manufacturing exporters.
	[2]	2020	Survey on green logistics management practices direct and indirect effects on Chinese industries.
Shipping industry			
	[23]	2020	A qualitative case study of perceptions of logistics buyers (shippers) and logistics service providers about green logistics practices.
Road Freight industry			
	[24]	2015	Survey on challenges to adopting green logistics in road freight industry.
Electronics and platforms			
	[25]	2020	Survey on Korean workers intention to use green logistics platforms.
	[26]	2010	Survey on Taiwanese electronic manufacturing businesses capability towards green supply chain management.
Fashion industry			
	[27]	2012	Survey on factors affecting Hong Kong consumers eco-fashion decisions.
	[8]	2014	Survey on factors affecting American adults eco-friendly apparel purchase intentions.
Pharmaceutical industry	[28]	2020	Study on the effects of governmental subsidy strategies on the adoption of green logistics by the pharmaceutical industry.
General	[4]	2013	Survey on factor affecting Chinese rural consumers ecological consumption behaviour.
	[7]	2013	Survey on customer involvement in green supply chain.

The novelty of this study can be summarised as follows. First, there have been few studies conducted on consumers’ willingness to participate in the green logistics of CEP firms, particularly in China. This study fills this gap and conducts an online survey of Chinese consumers’ willingness to participate in CEP firms’ green logistics. Second, based on the literature and expert opinion, this study built a comprehensive, structured questionnaire to assess the impact of various relevant variables and, subsequently, latent factors on Chinese consumers’ willingness to participate in the industry’s green logistics. Future studies on this subject may adopt or amend this questionnaire, saving time and effort. Third, the study addresses some of the technical issues related to survey-based studies and provides a technically sound statistical approach. The issues include: (1) Instead of presenting principal components analysis (PCA) as exploratory factor analysis (EFA) [29], this study distinguishes between the two and conducts EFA using principal axis extraction factor (PFA) instead of PCA. (2) During this century, many issues have increasingly been raised regarding factor analysis of questionnaire data, which is little known in the applied social sciences [30]. This is because survey data are typically ordinal, violating the assumptions of most statistical methods, and are thus often factor analysed inappropriately [31]. There is a growing consensus that the best approach to analysing categorical variables (with few categories) is through diagonally weighted least squares (DWLS) [32]. To address the categorical nature of data, a DWLS estimator that is based on a polychoric correlation matrix has become the most popular method [33]. However, many recent survey studies, particularly on the topic of green logistics, do not cater to the ordinal nature of Likert-scale type data [16, 17, 26, 29], and use factor analysis methods that are primarily designed for continuous data, and not for Likert-scale type ordinal survey data. This study performs confirmatory factor analysis (CFA) under a DWLS approach, which is the most popular approach to the factor analysis of Likert scale-based ordinal data. (3) Multiple linear regression analysis is generally applied to test hypotheses or measure the influence of different independent items on the dependent variable in survey studies [6, 24, 27]. However, where the dependent variable is measured on a Likert scale, simple linear regression is inappropriate [34]; instead, ordinal linear regression (ODR), Multinomial logistic regression (MLR), or collapsing the dependent variable into two levels and conducting binary logistic regression should be adopted [34]. Both ODR and MLR are used for categorical outcomes in at least three categories [35]. However, compared to ODR, MLR is bounded by fewer restrictions [36], while the MLR test in SPSS (Version 23) groups responses to all independent variables according to the categories of the dependent variable (for example, customers are grouped according to their high or low levels of willingness), which makes it easy to interpret the results. This technically correct estimation of survey results may help future studies produce technically sound papers, particularly in the field of green logistics.

## 2. Methodology

### 2.1. Initial hypothesis development

A hypothesis is an educated guess because it is based on previous research or knowledge known about a subject [37]. A well-defined and precise research question and research hypothesis are likely to help guide the direction and scope of analysis and how data are collected [38]. For the purpose of developing the initial hypothesis, the authors relied on previous studies (both in Chinese and English) and expert colleagues’ opinions. There is always the possibility of a Type I error, which occurs when a null hypothesis is rejected when it is true, and a Type II error, which occurs when a false null hypothesis is not rejected. To minimise the risk of making a Type I error, a lower significance level (*α*) can be used, while the risk of making a Type II error can be minimised by ensuring that an experiment has higher statistical power [39]. To minimise the possibility of making a Type I error during the directional hypothesis testing (which is performed later via correlation analysis), the significance level is set to the popular *α* = 0.05; the null hypothesis is not rejected above this value. SPSS (version 23) was used to estimate the statistical power between all the variables at the *α* = 0.05 level of significance (specifically, the ‘repeated measures’ option in SPSS (version 23) was used to estimate the statistical power). The statistical power of this study is 100% (1.00), which means that, at a significance level of *α* = 0.05, there is no likelihood of encountering a Type II error. The hypotheses and their selection criteria follow below.

*H1(Payment)*: *Positive relationship between payment for green logistics and Chinese consumers’ willingness to participate in the green logistics of the CEP business (CWPGL)*.

Payment or cost is one of the most critical factors that guides consumer green purchasing decisions [7, 27]. Güner and Coşkun have claimed that green logistics costs are among the major obstacles to the implementation of green logistics by businesses [40]. Chan and Wong have suggested that the price premium affects the eco-fashion choices of Hong Kong customers [27]. Hao et al. found that the green package price was an important factor in Chinese consumers’ inclination to green packaging [17]. A customer’s ability to pay for green logistics is therefore an important factor in CWPGL.

*H2 (Support)*: *Positive relationship between support for express delivery companies in developing green logistics and CWPGL*.

Motivation is among the main factors in the development of environmentally friendly products [41]. It can therefore be argued that, without customer support (motivation), it is difficult for businesses to develop green practices. Accordingly, this study assumes that there is a positive relationship between Chinese consumer support for the development of green logistics by CEP companies and CWPGL.

*H3 (Time spent)*: *Positive relationship between time spent on understanding the operation of green logistics in express enterprises and CWPGL*.

Customer knowledge has been shown to be positively correlated with green business practise [7, 42]. Indeed, 18 recent studies proposed a relationship between knowledge and green procurement, while 15 studies showed a positive relationship between knowledge and consumer willingness to purchase green [43]. Specifically, Wang et al. showed that environmental knowledge played a key role in guiding China’s rural consumers through sustainable consumption habits [4]. Furthermore, Zhao et al. found that environmental knowledge had a significant impact on the green consumer behaviour of Chinese consumers [6]. In this study, we also considered this fact, and conjectured that the time spent by customers learning about green logistics was positively related to CWPGL.

*H4 (Environmental consideration)*: *Positive relationship between environmental protection consideration and CWPGL*.

Wang et al. found a positive relationship between the concerns of Chinese customers about the environment and sustainable consumption patterns [4]. Specifically, Jazairy reasoned that buyer’s green concerns played a key role in their ‘green logistics purchasing’ [23]. Cowan and Kinley showed that environmental concern was among the key variables affecting the eco-friendly apparels purchase intentions of American adults [8]. Similarly, this study hypothesises a positive relationship between Chinese customers’ environmental concerns and CWPGL.

*H5 (Reuse)*: *Positive relationship between the reuse of old parcel packages and CWPGL*.

Multi-national companies, such as Starbucks, use tactics such as the re-use of coffee mugs to promote customer participation in green development [44]. Specifically, findings by Hao et al. suggest that reusability of packages can promote the willingness of customers to use green packaging in China [17]. Alternatively, the same principle of reusing old parcel packages (boxes) may have a positive impact on CWPGL.

*H6 (Shared boxes)*: *Positive relationship between the use of shared parcel boxes and CWPGL*.

Sharing parcel boxes means fewer boxes used to ship parcels to the same neighbourhoods (vicinities), which can reduce not only the cost of packaging, but also the environmental footprint of the packaging operations of express companies. Therefore, based on expert opinion, the use of shared packages can be a good estimate for CWPGL.

*H7 (Recycling)*: *Positive relationship between following the recycling instructions on parcel packages and CWPGL*.

Recycling and reuse efforts can have a positive impact on customers’ involvement in green logistics [7]. Some services industries, such as hotels, encourage their customers to adopt recycling practices to promote green procurement [44]. Similarly, it is assumed that consumer recycling practices have a positive impact on CWPGL.

*H8 (Positive response)*: *Positive relationship between people’s positive response and CWPGL*.

Studies have shown that promoting people’s response in green logistics by organisations can have a positive impact on the development of green logistics [7, 17]. Accordingly, this study assumes that Chinese people’s positive response is a good indicator of CWPGL.

*H9 (Shared pickup locations)*: *Positive relationship between parcel pickups at public (shared) places and CWPGL*.

The concept of common public pick-up points for express parcels is growing worldwide. For example, in most countries, universal service providers (USPs) have installed parcel lockers, typically located in high-frequency locations, providing consumers with the ability to receive parcels within convenient timeframes [45]. Establishing common public pick-up points can not only save money, but it also reduces the need for home delivery and pick-up, which, in turn, can reduce CO_2_ emissions from the transport activities of express companies. Researchers, therefore, focus on optimum locations for common public pick-up points, particularly for China [46]. Accordingly, this is an important measure of CWPGL.

*H10 (Raising fees) Positive relationship between encouraging raising charges (fees) by express companies and CWPGL*.

Price is one of the biggest barriers to the adoption of green logistics by different companies. Therefore, experts suggest that when customers encourage logistics companies to increase their fees to pay for green operations, it represents another dimension through which the price barrier to the adoption of green logistics by express companies can be removed, and can serve as a good measure of CWPGL.

*H11 (Community help)*: *Positive relationship between community help (guidance) and CWPGL*.

A community plays an important role in motivating climate-friendly behaviour [47]. Li et al.’s findings suggest that external support factors play a key role in Chinese companies’ willingness to engage in green coal logistics [16]. Hao et al.’s results indicated that ‘Impacts from Others’ played a role in the willingness of Chinese consumers to pay for green packaging [17]. This study postulates that community support can have a positive impact on CWPGL.

*H12 (Corporate strategy)*: *Positive relationship between encouraging inclusion of green logistics in corporate strategy and CWPGL*.

Pillay and Mbhele showed that company strategy was an important factor in the adoption of green logistics in the freight industry [24]. Accordingly, this study postulates that Chinese customers’ perception of the role (importance) of green logistics in corporate strategy is positively linked to CWPGL.

*H13 (*Ecological mode of working*)*: *Positive relationship between an ecological mode of working (reduced material*, *inner cushions*, *tape*, *etc*.*) and CWPGL*.

Lai and Wong showed that environmental and operational efficiencies were vital to the adoption of green logistics management by Chinese manufacturers [22]. Shang et al. found that reducing waste, minimum energy use, etc. were key factors in consumers’ willingness to pay premium prices for green packaging [26]. Customers’ desire for an ecological mode of working in express companies is therefore a strong indication of CWPGL.

*H14 (Volunteering)*: *Positive relationship between volunteer work and CWPGL*.

Multi-national companies, such as Starbucks and Kuoni, and government agencies, such as the Thai Tourism Authority, focus on voluntary work to promote green procurement [44]. Pillay and Mbhele reported that voluntary transport of eco-friendly goods was an important factor in the adoption of green logistics by road freight companies [24]. Thus, it is hypothesised that volunteering to publicise green logistics by Chinese consumers has a positive relationship with CWPGL.

### 2.2. Sample strategy

A survey was conducted based on a convenience-sampling approach. Online surveys are considered to be more convenient, faster, more accurate, cheaper, quicker and more flexible than conventional sources [48]. Various public and private groups in popular messaging applications in China, such as WeChat and QQ, were used to convey survey questions through QR (Quick Response) codes. The groups were chosen to cover respondents from diverse demographic backgrounds. The research was approved by the institutional review board of the Xuzhou University of Technology. In addition, an informed consent form embedded in the online survey informed the participants about the survey: the introduction (voluntary nature and background), purpose, procedure, risks, benefits, and privacy protection. The data were analysed anonymously, which meant that no personal identifiers would be included in the publication. Before participating in the survey, potential participants had to click a button indicating that they had read the consent information and had agreed to participate; minors had to have a signed parental/guardian consent form scanned and sent to the corresponding author via WeChat, QQ, or email.

The goal was to gather, at a minimum, 150 responses from the users of CEP services in China. With survey-based research, a minimum of 100 responses are required for marginal accuracy [49]. A QR code was posted into five QQ groups with membership numbers n = 56, 60, 45, 200 and 80, and three WeChat groups with the numbers n = 49, 36 and 100. A total of 159 responses were received, with a response rate of almost 25.4%. Of these 159 responses, four responses were unusable. Logistics and supply chain management surveys usually yield low response rates [26], with some survey studies reporting response rates of 18% and 17.1% [26]. In the context of our study, a response rate of 25.4% was considered acceptable: given the total population of China, the associated sample error for N = 155 is nearly ±8%.

The demographic structure of the sample is shown in Table 2. Of the 155 respondents, 52.9% were female, while 47.1% were male. Of the respondents, 65.2% were aged between 18 and 45 years. Most respondents had a bachelor’s degree (52.9%), followed by a master’s degree or higher (12.9%) and a middle school diploma (11.0%). Participants from rural areas constituted 35.5%, followed by city centres (30.3%) and towns (25.2%). The largest proportion of the participants had full-time jobs (27.7%), followed by full-time students (23.9%) and part-time students (18.7%). Some 34.2% of the participants belonged to the income bracket 1–9,999, while the second highest number (29.0%) indicated zero income status.

**Table 2:** Demographic profile of the CEP services consuming respondents (N = 155).

Category	N (Number)	Percentage
**Gender**		
**Female**	82	52.9
**Male**	73	47.1
**Age**		
**18**–**45**	101	65.2
**13**–**17**	16	10.3
**46**–**69**	16	10.3
**Less than 12 years**	12	7.7
**More than 69 years**	10	6.5
**Education**		
**Bachelor’s degree**	82	52.9
**Master’s degree or higher**	20	12.9
**Middle school**	17	11.0
**Primary school**	14	9.0
**Technical secondary school**	12	7.7
**Other**	10	6.5
**Residence**		
**Village**	55	35.5
**City centre**	47	30.3
**Town**	39	25.2
**Suburb**	14	9.0
**Employment status**		
**Full-time employment**	43	27.7
**Full-time student**	37	23.9
**Part-time student**	29	18.7
**Dependent (e.g. Children, Old age)**	18	11.6
**Other**	11	7.1
**Part-time employment**	9	5.8
**Unpaid (e.g. Volunteer)**	5	3.2
**Housewife**	3	1.9
**Income (RMB per year)**		
**1**–**9,999**	53	34.2
**0**	45	29.0
**100,000**–**149,999**	13	8.4
**50,000**–**74,999**	12	7.7
**25,000**–**49,999**	10	6.5
**75,000**–**99,999**	10	6.5
**10,000**–**24,999**	8	5.2
**More than 150,000**	4	2.6

* Response selection options for demographic-related questions are designed with China’s social and cultural norms in mind.

## 3. Data collection and analysis

### 3.1. Research questionnaire

A structured questionnaire was used to collect data online. The questionnaire was as simple and as concise as possible, designed for maximum participation by non-professionals from different demographic backgrounds. A five-point multiple-choice Likert scale from ‘Strongly negative’ (1) to ‘Strongly positive’ (5) was used to estimate the independent and dependent variables. The main questions asked and the answers to the optional questions are presented in S1 and S3 Tables. The S1 File contains Likert-scale data for the 155 valid responses collected in our survey.

### 3.2. Reliability

Cronbach’s alpha was the preferred tool to test the reliability of the survey questionnaire [16, 17, 50]. Reliability refers to the consistency and stability of measurement [16]. Values for Cronbach’s alpha, α, range from 0 to 1, where the value of zero indicates no correlation between items; as the correlation between items increases, the value of α approaches 1 [51]. In the literature, there is no single acceptable reliability criterion for the (Cronbach alpha) coefficient, α. For example, Li, Chen, and Wang consider an α value ≥ 0.7 to be a high reliability score, 0.35 ≤ α < 0.7 to be an acceptable reliability score, and an α value < 0.35 to be a low reliability score [16]. However, for Hao et al. and DeVellis, a score equal to or greater than 0.7 and less than 0.8 is acceptable, while a score equal to or greater than 0.8 and less than 0.9 is a good reliability score [17, 52]. Generally, a value of α < 0.5 is not acceptable [51]. Cronbach’s alpha coefficient value for all 15 items in this survey is 0.916, with N = 155 respondents. This score indicates the strong internal reliability of our survey questionnaire.

### 3.3. Validity

Validity is the ability of an instrument (e.g. a questionnaire) to measure what it is expected to [53]; that is, the degree to which an investigator calculated what they intended to measure [54]. Content, criterion, and construct-related validity are the three main types of validation [53]. For the content-related validity of the current instrument, the initial questionnaire mostly relied on related domestic (Chinese) and international literature, in addition to opinions of experts in the field of logistics. Finally, sampling bias was minimised by conducting random sampling in public and private groups via online open invitations, ensuring that all members from diverse demographic backgrounds had an equal probability of being selected. However, there is always the possibility of sampling error; therefore, a non-response bias test using the independent sample t-test was conducted in SPSS (version 23). The independent sample t-test was not accepted at p = 0.65 (95% CI = -0.54, 0.34), indicating that there was no statistically significant non-response bias in our sample. Section 5 presents the details of this non-response bias test and answers to some other perceived issues regarding the sampling strategy of our study.

Factor analysis is normally used to test the validity of a construct [16, 55]. It is a statistical approach that describes the relationship between many indicators or factors using a few variables, and represents much of the questionnaire data [16]. The Kaiser-Meyer-Olkin measure of sampling adequacy (KMO) and Bartlett’s test of sphericity are mostly used to check whether or not the data are appropriate for factor analysis [16, 17, 50]. SPSS (version 23) was used to conduct both the KMO test and Bartlett’s test of sphericity in this study. The KMO coefficient value for this study is 0.892, which is significantly higher than the acceptable value of 0.5 [17, 50, 56]. The null hypothesis for Bartlett’s test of sphericity is also rejected at a significance value of p < 0.001, which means that factor analysis is appropriate for the study. Table 3 presents the details of these two analyses.

**Table 3:** KMO and Bartlett’s test of sphericity.

**Kaiser-Meyer-Olkin Measure of Sampling Adequacy (KMO)**	0.892
**Bartlett’s Test of Sphericity**	
**Approx. Chi-Square**	1.18E+03
**Degree of freedom (df)**	91
**Significance (p)**	< 0.001

#### 3.3.1. EFA with PFA

S1 Appendix provides notes on the appropriateness and execution of EFA with PFA. Table 4 displays the pattern matrix of our EFA analysis with Cronbach alpha score for each new factor (construct). The factor loading cut-off value is set at 0.4 for this study; below this value, the factor loading score is considered irrelevant, since only EFA loading factor scores above 0.4 are considered stable [57]. Factor Number 1 consists of the largest number of 8 items, which directly or indirectly relate to the consumers’ economy: payment, time spent, environmental considerations in daily life, the reuse of old boxes, the use of shared boxes, recycling, positive response, and an increase in the fee for developing green logistics all involve economics in one way or another. The second factor is customers’ willingness to engage in CEP companies’ green logistics operations. It includes support for green logistics operations, encouraging the inclusion of green logistics in CEP companies’ corporate strategies, and promoting an ecological mode of working. Factor 3 comprises mainly social items (drivers), such as shared pick-up locations, community help, and volunteering to publicise green logistics.

**Table 4:** Result of EFA.

Items	Factor loadings		
	1	2	3
**Economic factor**			
**Payment**	0.787		
**Time spent**	0.508		
**Environmental consideration**	0.641		
**Reuse**	0.751		
**Shared boxes**	0.459		
**Recycling**	0.454		
**Positive response**	0.462		
**Raising fee**	0.650		
**Operational factor**			
**Support**		0.513	
**Corporate strategy**		0.717	
**Ecological working**		0.868	
**Social factor**			
**Shared pickup locations**			0.607
**Community help**			0.474
**Volunteering**			0.909
**Eigen value**	6.65	1.56	1.12
**Total Variance explained (%)**	44.55	8.38	5.31
**Cronbach alpha**	0.887	0.797	0.769

^a.^ Presents the pattern matrix of PFA. ^b.^ Extraction Method: Principal Axis Factoring. ^c.^ Rotation Method: Oblimin with Kaiser Normalisation.

#### 3.3.2. CFA with DWLS

CFA is a statistical method that is used to verify the factor structure of a set of observed variables [58]. CFA allows an investigator to test the hypothesis that the observed variables and their underlying latent constructs (factors) are related [58]. CFA and EFA are related methods; however, in EFA, data are simply discussed, and details on the number of latent variables (factors) required to represent the data are provided [59]. By contrast, CFA is a method used to affirm or refute a measurement model [59]. For CFA, this study uses a DWLS estimator, which is strongly suggested for Likert scale-based ordinal data. This study uses the latent variable analysis (lavaan) package that is freely available to R users [60]. Following the standard process, the lavaan package sets the path coefficient for one observed variable to ‘1’ for each latent factor (see [61]). S4 Table provides the items and factor loadings (covariances), standard errors, and z-test scores of our model.

Model fit indices can represent how well the model fits the data [61]. Traditionally, the chi-squared test has been used to assess the goodness of fit of a model; however, it has several shortcomings [61]. For example, the chi-squared model depends on sample size: models with larger samples tend to have more significant results than those with smaller samples [61]. Consequently, researchers currently use different model fit indices. In this study, different goodness-of-fit indices and their accepted standards are presented as per the minimum criteria from [16] and [62] for the CFA. These include the chi-squared test score (CMIN (X^2^)), chi-squared ratio or chi-squared to degree of freedom ratio (CMIN/DF (X^2^/DF)), goodness-of-fit (GFI), adjusted GFI (AGFI), non-normed fit index (NNFI, also referred to as TLI), comparative fit index (CFI), root mean square residual (RMR (standardised)), and root mean square error of approximation (RMSEA). Table 5 presents the details. For the CMIN (X^2^), a probability p ≥ 0.05 is desirable. The null hypothesis is not rejected at p < 0.001, primarily due to the medium-sized sample (N = 155) used in the study. When 100 ≤ N ≤ 200, a sample is considered to be of medium size in many cases [61, 63]. In our case, the p value is influenced by the inherent weakness of the chi-squared test, i.e. the dependence on sample size. However, the CMIN/DF of 2.2 is within the standard acceptable range, i.e. between 1 and 3. The values of both the GFI (0.986) and the AGFI (0.974) are within the accepted standards of ≥ 0.85 and ≥ 0.80, respectively. The NNFI (TLI) is less affected by sample size [64] than the normed fit index (NFI): its value is 0.988, which is greater than the minimum recommended value of 0.90. Compared to the NFI and NNFI, the CFI is less affected by sample size [64], and has a value of 0.990, which is greater than the minimum suggested value of 0.90. The RMR values are dependent on the scale of input variables and are difficult to interpret; therefore, standardised RMR (SRMR) values are recommended [61]. The standardised RMR score is 0.08, which is within the limit of 0.08.

**Table 5:** Goodness-of-model fit indices.

Fit indices	Value	Accepted standard
**CMIN (X**^**2**^**)**	164.071 ^a^	The smaller is good
**CMIN/DF (X**^**2**^**/DF)**	2.2	>1 and <3
**GFI**	0.986	≥0.85
**AGFI**	0.974	≥0.80
**NNFI (TLI)**	0.988	≥0.90
**CFI**	0.990	≥0.90
**Standardised RMR (SRMR)**	0.08	≤0.08
**RMSEA**	0.089	≤0.08

^a.^ Here, the value of X^2^ is significantly smaller than the value (663.853 after modification) reported in some recent related studies (see, for example, [16]).

**Fig 1:**
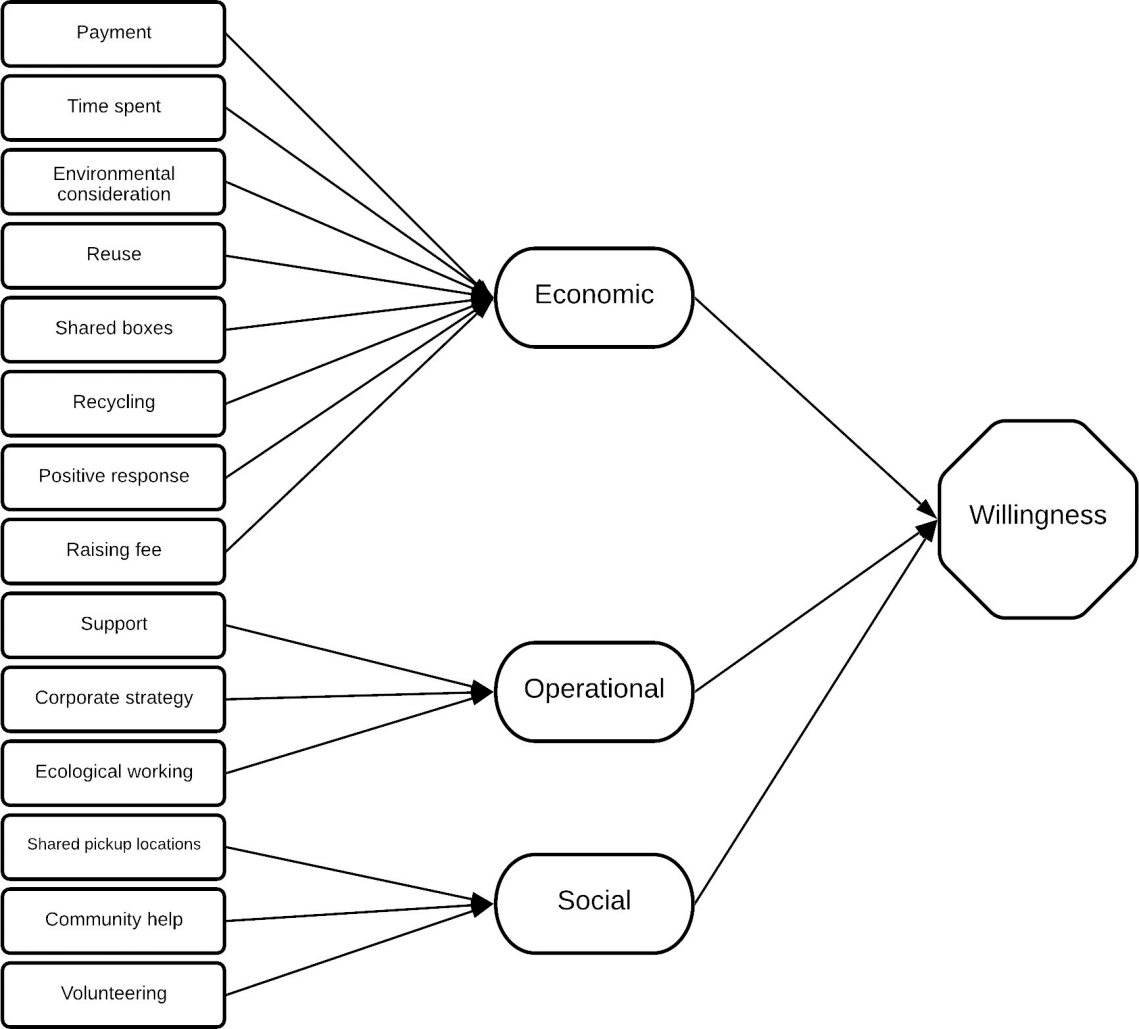
The validated model of this study. EFA and CFA are used to validate the model.

An RMSEA represents the value of unexplained variance or residuals. The RMSEA value is 0.089, which is slightly higher than the acceptable limit of 0.08. However, other popular indices, such as the CFI, indicate an excellent model fit. The RMSEA and CFI are amongst the most popular goodness-of-model fit indices; when the RMSEA and CFI are inconsistent, researchers should not automatically disregard the model, merely because one index fails to meet the cut-off, nor should they retain the model by reporting only the good-fit index [65]. Instead, researchers should investigate why the indices disagree, and the implications of the disagreement [65]. The RMSEA for models with medium-sized samples (as in our case) and small degrees of freedom mostly falsely indicate a poor model fit [66]. Overall, for the current study, most of the goodness-of-model fit indices indicate that the model is a good fit, and is valid for investigating the research question, i.e. Chinese consumers’ willingness to participate in the green logistics of express companies. Fig 1 presents the validated model of this study.

## 4. Results

### 4.1. Descriptive statistics

**Fig 2:**
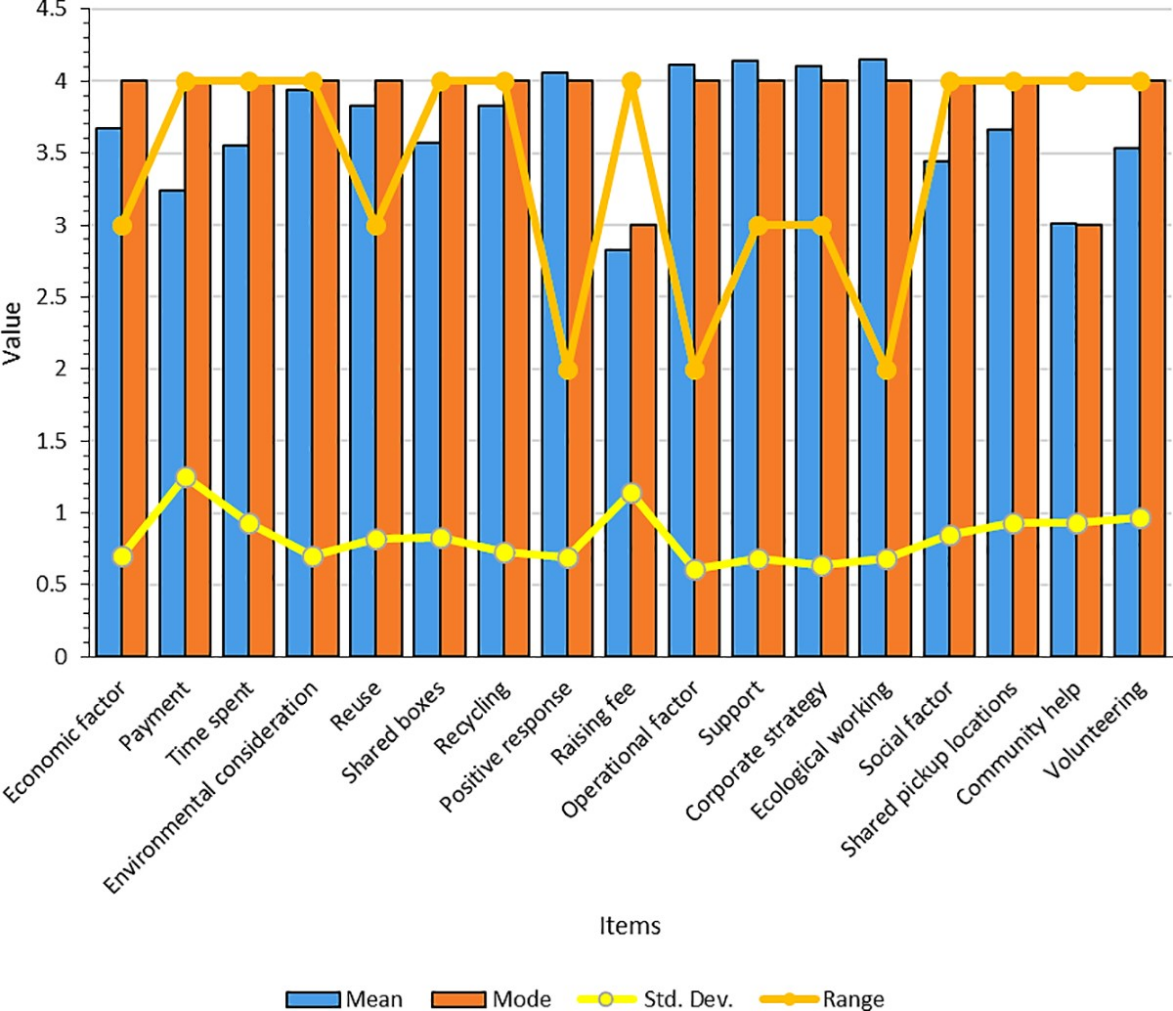
Descriptive statistics. Factor scores (values) are calculated by averaging the values of variables that belong to the same factor. This is a more accurate approach, compared to using the actual factor scores (z-scores) generated [67], because the mean of z-scores is zero and the standard deviation is 1. The mean scores correlate perfectly with the actual factor scores, without suffering from the weaknesses of the latter [67].

Fig 2 presents the descriptive statistics of this study. Of the three factors, the operational factor (N = 155) has the highest mean value of 4.11. The most frequent value (mode) is 4, while the factor values range between 3 and 5. The item, ecological working (N = 155), from the operational factor, has the largest mean score of 4.15, with a modal value of 4 and a range of 3–5. The economic factor (N = 155) has the second largest mean score of 3.67, a modal value of 4, while the range is between 2 and 5. Under the economic factor, the positive response (N = 155) item has the highest mean score of 4.06, a modal value of 4, and ranges between 3 and 5. Finally, the social factor (N = 155) has an avergae value of 3.44, a modal vlue of 4, and a range of 1–5. Under the social factor, the shared pickup (N = 155) locations have the highest mean score of 3.66, a modal value equal to 4, and range between 1 and 5.

### 4.2. Correlation analysis

**Fig 3:**
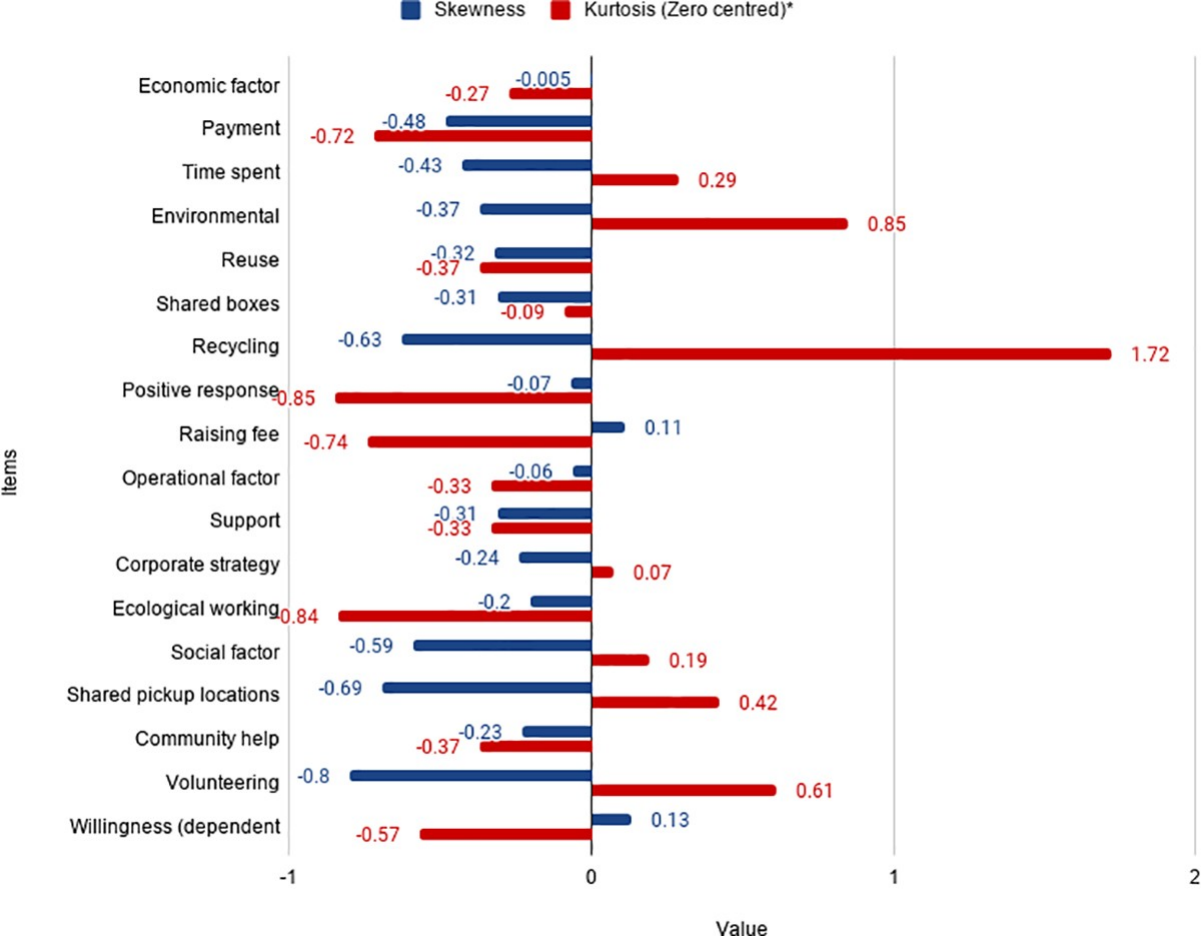
Skewness and kurtosis. *Zero-centred kurtosis is estimated by deducting a value of three from the usual kurtosis that has a normal distribution value of 3.

This study uses correlation analysis to test the hypothesis proposed by the research question, using validated data that have been tested for reliability. Correlation analysis helps us determine *(1)* whether there is a significant level of (directional) relationship between the dependent variable, on the one hand, and the independent variables and factors, on the other, and *(2)* whether or not the relationship direction is consistent with the hypothesised direction of the relationship. Researchers often use correlation coefficients to test the directional relationship between two variables [68]. Recent survey studies on related topics have similarly used correlation analysis to test hypotheses (see, for example, [6]). Pearson’s and Spearman’s correlation coefficients are commonly used for testing the directional relationship between dependent and independent hypothesis variables [68]. Pearson’s correlation can only be applied to non-normally distributed Likert-scale data (variables) when skewness and kurtosis are mostly less than 1, in absolute value terms [69]. Fig 3 shows that most items have skewness and kurtosis between -1 and 1. Therefore, Pearson’s correlation is used to test the hypothesis. The values of skewness and kurtosis (Zero centred) are estimated using SPSS (Version 23).

**Fig 4:**
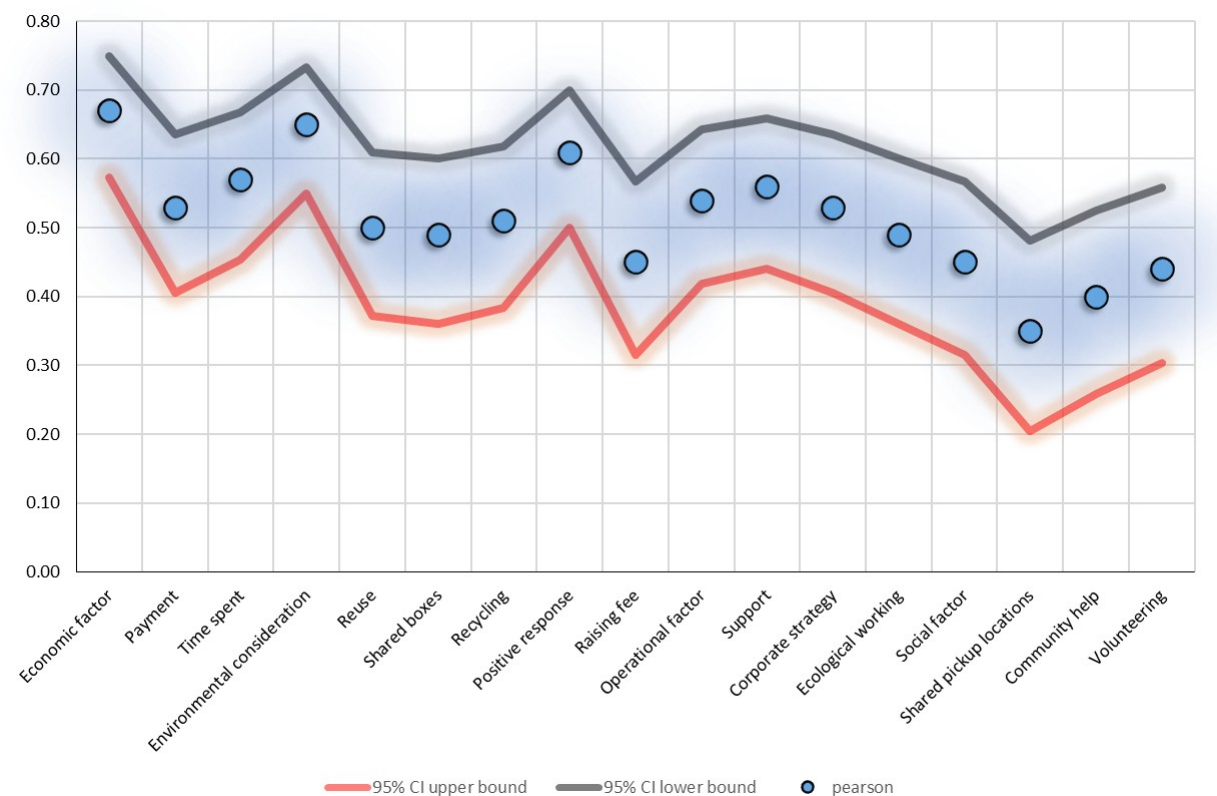
Pearson correlation analysis of the independent variables and latent factors versus the dependent variable. The correlation is significant at the α = 0.01 level (one- and two-tailed).

Pearson’s correlation coefficient values for the relationship between the independent variables and the dependent variable are shown in Fig 4. Pearson’s correlation coefficient and both one- and two-tailed tests of significance are estimated using SPSS (version 23). The 95% confidence intervals are estimated using the *how2stats* online calculator for correlations and confidence intervals [70]. In both the one- and the two-tailed tests, all the independent variables and latent factors showed significance at p < 0.001. The correlation value varies between 1 and -1. Conventionally, the following scheme is applied for the absolute value of the correlation coefficient, R: 0.00 ≥ R < 0.10 is considered to be insignificant (unacceptable), 0.10 ≥ R < 0.4 weak, 0.4 ≥ R < 0.7 moderate, 0.7 ≥ R < 0.9 strong, and ≥ 0.9 very strong [68]. To reject the null hypothesis (H0: R ≤ 0.09), meaning a negative or an insignificant relationship between the dependent variable (willingness) and the independent items, the value of R should be > 0.09. As shown in Fig 4, Pearson’s correlation coefficients for all the items are significantly greater than 0.09, indicating that we can reject the null hypothesis in favour of the alternative hypothesis (Ha: R > 0.09), which states that there is a significant positive relationship between the dependent and independent items. The economic factor has the highest R coefficient of 0.67 (95% CI = 0.57, 0.75; p < 0.001; N = 155), followed by operational (R = 0.54; 95% CI = 0.42, 0.64; p < 0.001; N = 155) and social (R = 0.45; 95% CI = 0.32, 0.57; p < 0.001; N = 155) factors. Most items indicate moderate correlation. The items with the largest R values are environmental consideration (R = 0.65; 95% CI = 0.55, 0.73; p < 0.001; N = 155), positive response (R = 0.61; 95% CI = 0.50, 0.70; p < 0.001; N = 155), and time spent (R = 0.57; 95% CI = 0.45, 0.67; p < 0.001; N = 155). The shared location item from the social factor has the lowest correlation value of 0.35 (95% CI = 0.20, 0.48; p < 0.001; N = 155).

### 4.3. MLR

MLR is used to predict the categorical placement or likelihood of a dependent variable’s membership based on several independent variables [71]. Independent variables can be binary, intervals, or ratios, scaled for MLR analysis [71]. For the current study, an MLR analysis was conducted between the dependent variable, willingness, and the independent variables, using SPSS (version 23). The reference category was designated as the ‘last category’ in an ascending order of the categories. To conduct the MLR analysis, the default ‘main-effects’ model was chosen. For the convergence criteria and model options, such as dispersion scale and stepwise options, SPSS (version 23) defaults were used.

The first step is to determine whether the final model is superior to the null, i.e. whether the variables included in the final model statistically improve the model over the intercept-only model with X = 0. In this case, the final model should have a value of p < 0.05 to reject the null model. As can be seen from Table 6, the final model is significant at p < 0.001 (N = 155), which means that the final model predicts the dependent (outcome) variable better than the zero model. Other fit indices, such as Pearson’s chi-squared test and the deviance chi-squared test, should have a statistical value of p > 0.05 and, preferably, smaller chi-squared values for better fit. Both these model-fit statistics have p-values above 0.05 (N = 155), and not very large chi-squared values, indicating a good model fit. Pseudo R-Squared values for Cox and Snell, Nagelkerke, and McFadden are also within a good range of 0.66–0.85. S1 Appendix contains information about the statistical significance (p) and confidence intervals (CIs) associated with the study’s individual variables for the MLR analysis.

**Table 6:** Model fit statistics for multinomial regression.

Item	Chi-Square	df	Sig. (p)
**Model Fitting Information**			
**Final model (Likelihood Ratio Tests)**	207.301	144	< 0.001
**Goodness-of-Fit**			
**Pearson**	85.159	276	1.00
**Deviance**	103.478	276	1.00
**Pseudo R-Square**			
**Cox and Snell**	.737		
**Nagelkerke**	.850		
**McFadden**	.661		

**Fig 5:**
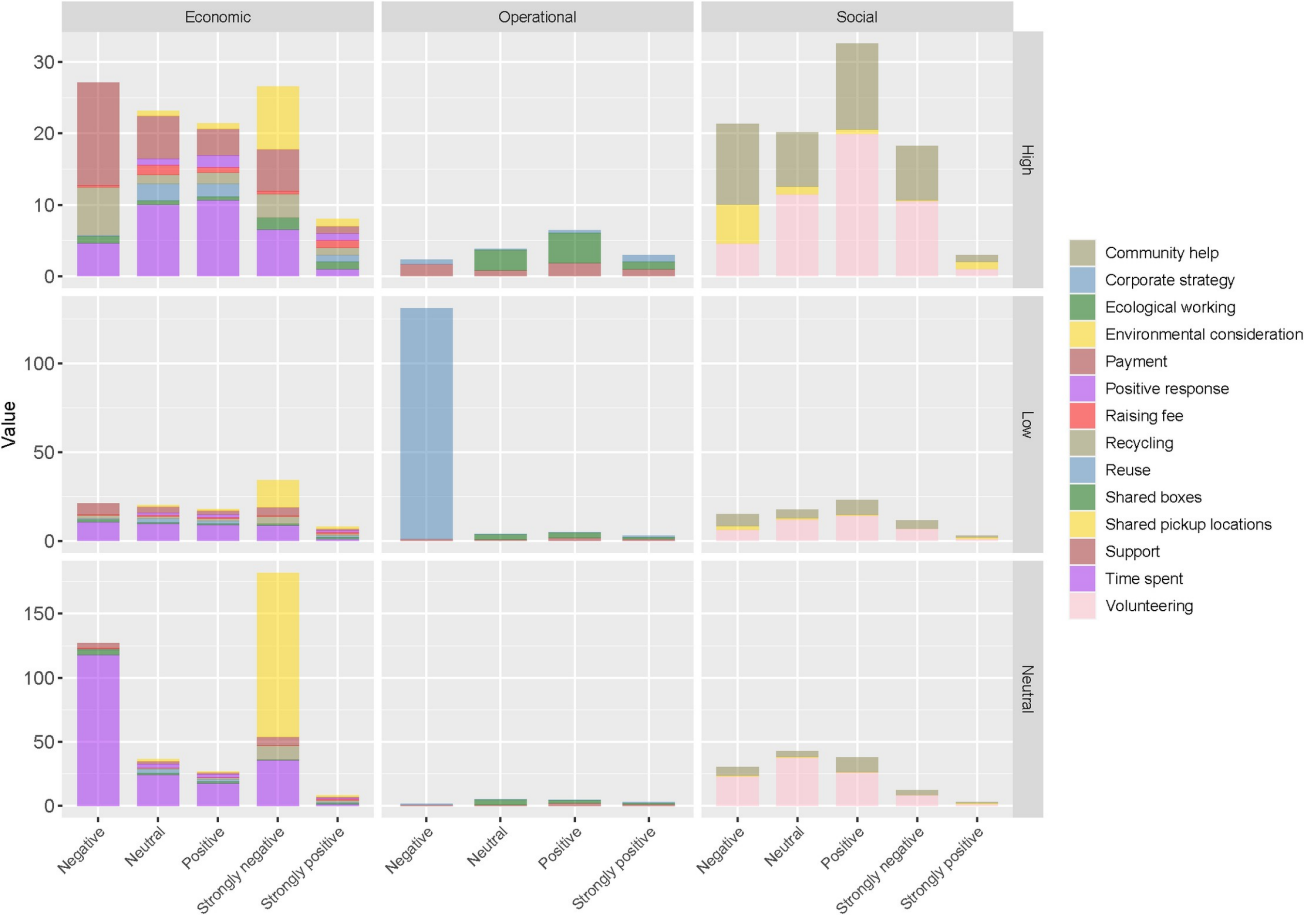
Multinomial regression analysis results. The figure depicts the Exp (B) odds of selection. The vertical columns classify customers based on their degree of willingness (the dependent variable), while the horizontal rows categorise the items according to latent factors. Corporate strategy’s maximum odd value under the operational factor has been re-scaled for comparison purposes.

The reference dependent variable in our MLR analysis is the ‘very high’ willingness group, while the ‘strong positive’ willingness group serves as the reference response for the independent items. To facilitate interpretation, Fig 5 presents only the Exp (B) column, which represents the odds of a customer selecting a category over the reference category. An Exp (B) value of 1 is assigned to the reference independent variable. To keep things simple, only the maximum multinomial odds of belonging to a particular group are discussed here for the independent variable. As the Exp (B) value for the various independent variable scores decreases, the likelihood of customers falling into the reference group increases relative to that of remaining in the current group. Consequently, the odds of being in the reference dependent category of ‘extremely high’ willingness are not compared to those of the independent variable, i.e. instances in which the likelihood of belonging to a particular group is lower than that of belonging to the reference category.

The ‘low’ willingness group, with only 2/8 items with high odds of positive and strong positive scores, and 1/8 with high odds of a neutral score, has high odds of unwillingness to directly or indirectly participate in the economic factor; however, having 2/3 items with high odds of a positive score, this group is more likely to participate in both operational and social factors. For most items, customers from the ‘moderate’ or ‘neutral’ willingness group unsurprisingly have maximum odds of having negative or neutral scores. Again, the most reluctant factor is the economic factor, with 3/8 items with maximum odds of neutral scores, and ‘others’, with a negative or strongly negative score. With one item positive, one negative, and one neutral, this group will most likely be somewhat indifferent, i.e. neutral to the operational factor. With 1/3 items having a maximum likelihood of positive scores, and 2/3 with a maximum likelihood of neutral scores, this group is most likely to participate in or encourage the social factor. Last, the ‘high’ willingness group, with 2/8 items with maximum odds for a positive score, and 1/8 with a neutral score, has a high likelihood to not participate in or encourage the economic factor. With 2/3 having maximum odds of a positive score, and 1/3 items with a strongly positive score, the ‘high’ willingness group is most likely to encourage or participate in the operational factor. Of the three groups, the ‘high’ willingness is the only group with high odds of selecting all items positively from a factor, i.e. the operational factor. Finally, this group, with 2/3 items with high odds of a positive score, is also likely to participate in or promote the social factor. In summary, all three groups are highly likely to not participate in the economic factor, while participation in the operational and social factors is more likely. Specifically, the ‘high’ willingness group has a very high likelihood of engaging in or encouraging the operational factor.

## 5. Discussion

Consumer demand has a huge impact on the industrial environmental footprint [72]. Consequently, it is critical to comprehend consumers’ willingness to participate in an industry’s green operations. Specifically, in China, household (consumer) demand has been shown to have a substantial impact on industrial carbon emissions [73–77]. Logistics operations are estimated to account for 8% of worldwide CO_2_ emissions [78]. Additionally, the logistics industry is a significant source of air pollution [78]. In comparison to many other sectors, freight emissions continue to grow [78]. The CEP industry, whose core business is based on logistics, has a major role in a country’s carbon footprint [1]. However, consumers’ willingness to participate in the green logistics of CEP companies has not been well studied. This study filled this research gap by conducting an online survey of 155 Chinese consumers from diversified demographic backgrounds. This presentation of the key items and latent factors that influence consumer willingness to participate in green logistics can aid in the development of consumption-side policies and subsequent action plans, while keeping in mind the most important motivators and/or barriers to consumer participation in the CEP industry’s green logistics, particularly for Chinese CEP consumers.

Furthermore, the study clarified the use of some of the common techniques in survey research. The study differentiated between PCA and EFA, a distinction that is often neglected in related studies [29]. Some survey studies do not consider the ordinal nature of Likert scale-based survey data when performing CFA [16, 17, 26, 29]. The study used the DWLS approach, which is one of the most appropriate methods for the CFA of categorical data. MLR analysis was used in this study, which is more appropriate for ordinal data, instead of the usual multiple regression analysis [6, 24, 27]. Furthermore, in the literature, Pearson correlation analysis has been used without testing its appropriateness for specific survey studies [6]. However, skewness and kurtosis tests were performed, in this study, to justify the use of Pearson’s correlation on non-normally distributed Likert-scale data.

The EFA and CFA helped us to narrow down the scope of the study to three main latent factors: the economic (financial), operational, and social factors. The economic factor is defined by the items (independent variables) that are more or less related to the monetary effects of the adoption of green logistics practices by the CEP industry. The operational factor comprises items that do not have any direct or indirect financial or economic effect on CEP consumers, but instead indicate consumers’ preference for the incorporation of green logistics in the operations of the CEP industry. Finally, the social factor comprises factors that are more or less related to the willingness of consumers to participate in social activities that are related to the promotion of the green logistics of the CEP industry. The results supported the hypothesis, indicating that all three latent factors and their corresponding items had a statistically significant (one- and two-tailed) positive correlation with the dependent variable (i.e. consumer willingness). In other words, where the value of a latent factor, influenced by its member items (independent variable), increases or decreases, the consumers’ willingness to participate in CEP firms’ green logistics is expected to change in the same direction. Furthermore, the results of the multinomial regression analysis revealed that Chinese consumers were highly likely to show a low willingness to participate in most of the activities (items) related to economic factors. In simple terms, the consumers are unlikely to participate in activities (items) falling under the economic factor, while they are highly likely to participate in both the operational and the social factors. Although, as discussed in Section 1, there is scant literature on consumers’ willingness to participate in CEP companies’ green logistics, there are some studies on consumers’ willingness, attitudes, intentions, and behaviour towards green logistics and green purchases, generally. Most of these studies do not specifically categorise the items that affect consumers’ willingness (attitude or behaviour) to participate in green logistics into the three main latent factors, i.e. the economic, social and operational factors, as defined in this study. However, as mentioned in Subsection 2.1, different studies have presented most of the items falling under these latent factors, which further emphasises the relevance of the items (independent variables) selected for the purpose of understanding the Chinese consumers’ willingness to participate in the green logistics of the CEP industry. More specifically, the economic items of payment [7, 17, 27, 40], time spent [4, 6, 7, 42, 43], environmental consideration [4, 8, 23], reuse [17, 44], recycling [7, 44], and positive response [7, 17] have been considered in separate, but related, studies in different green logistics and purchase contexts. Similarly, the operational items of support [41], corporate strategy [24] and ecological mode of working [22, 26], and the social items of shared pickup locations [45, 46], community help [16, 17, 47], and volunteering [24, 44] have also been shown to affect the adoption of green logistics by different companies.

It is vital for government policy makers to not only devise environmental laws and policy regulations, but also to enforce the adoption of green logistics by different companies. Meanwhile, the government should provide monetary and non-monetary incentives to companies practising green logistics. The government’s subsidy strategy for green logistics plays a vital role in the adoption of green logistics by businesses [28]. Therefore, it is recommended that the government should give monetary incentives to CEP companies that adopt green logistics, such as tax reductions and subsidies, to reduce the cost of adopting green logistics. However, encouraging consumer participation or increasing consumer willingness to support green logistics ultimately depends on consumer-oriented actions taken by firms practising or planning to practise green logistics. Particularly, as per our findings, there is a high likelihood that Chinese consumers will not participate in key green logistics activities that directly or indirectly involve economics (money) or monetary incentives. For example, the items for payment and raising fees directly involve the price tag of green logistics that affect consumers. Here, the CEP companies should ensure that the minimum additional costs of green logistics are transferred to the final consumers. Similarly, the time spent on understanding the operation of green logistics, positive response to the development of green logistics, and environmental considerations also indirectly affect the economics of the consumer. Therefore, the environmental benefits of green logistics should be propagated to the CEP consumer via advertisements, social media, direct interactions, etc. This is because previous studies have shown that consumers’ ‘environmental knowledge’ and ‘preferences’ play an important role in their willingness to pay more for green products [79]. Reusing, sharing boxes, and recycling, on the other hand, can be used to provide monetary incentives to consumers. Currently, most CEP companies do not provide any such monetary incentives to consumers who are willing to use old boxes, share delivery boxes with other consumers, or recycle boxes as per the instructions. This will not only encourage consumers to participate in reusing, sharing boxes, and recycling, but it can also save money for the CEP companies by reducing the volume of the expensive procurement of new boxes and raw materials via the lower final consumer demand for the new boxes (through reuse and sharing) and for raw materials by recycling. The monetary incentive policy, particularly for recycling, has been demonstrated to be a viable option for consumers. Some companies encourage consumers to resend garbage back to them through monetary incentives (in different ways) to the consumers [80]. Thus, a win-win strategy for the adoption of green logistics by the CEP industry, generally, and for the Chinese CEP industry, particularly, can be devised. All key stakeholders in CEP companies’ green logistics, such as environmental policymakers (government), the CEP industry, and CEP consumers, can gain various benefits.

Studies have shown that consumer motivation and emotions towards green products and services play a critical role in the adoption of green operations by businesses [81]. Although the likelihood of Chinese consumer’s participation in the operational and social factors is high, these factors cannot be ignored in the promotion of the Chinese consumers’ willingness to participate in the green logistics of the CEP companies. This is because these factors can have a positive effect on the both the CEP companies and the consumers’ participation in green logistics. Particularly, the consumers’ support, demand for the greening of the operations (ecological working mode), and demand for the inclusion of green logistics in the CEP company’s corporate strategy can persuade CEP companies to consider green logistics not only in their daily operations, but also in their company strategy. According to recent evidence, businesses are increasingly compelled to incorporate environmental protection strategies into their business strategies to remain competitive [82]. Similarly, social factors, such as community help, shared pickup locations, and volunteering can be further promoted, especially since most Chinese consumers live in multi-story residential buildings and gated housing societies. The community leaders in these small societies and residential apartments can be educated and motivated to propagate the importance of CEP green logistics to the community members. Shared pickup locations can be promoted as community interaction and recreation centres, for them to become more attractive, compared to home pickups. Finally, volunteers should be awarded certificates, gifts, and badges to improve the rate at which people volunteer to promote participation in CEP companies’ green logistics amongst Chinese consumers.

### 5.1. Limitations and future research

In this study, the authors used an online survey, which is unquestionably a more convenient, robust, and cost-effective method of surveying people. However, this type of survey has several limitations, for instance, the inability to reach certain populations, such as the elderly population and people with no educational background. However, China is a country with an extremely high proportion of smart phone users. Smart phones are owned by approximately 96% of the Chinese population, compared to a global average of only 90% [83]. Approximately 41% of Chinese people aged 61 years or older are frequent users of new technology, compared to a global average of only 16% [84]. More specifically, nearly 30% of Chinese people aged 50 years and older own a smartphone [85]. WeChat and QQ are the top two social media platforms in China [86]. Approximately 73.7% and 43.3% of Chinese mobile phone users frequently use these two applications [86], respectively. Over 19% of WeChat users are over 41 years old [87], while nearly 18% of QQ users are 40 years of age or older [88]. This demonstrates that a sizable portion of the Chinese population, both young and old, is familiar with and uses the WeChat and QQ platforms. Additionally, our respondents’ demographics indicated a somewhat similar trend, in which almost 17% of the respondents were aged 46 years or older. Furthermore, China has a very high literacy rate of 97% among its adult population [89], which implies that participants’ education level may not be a major barrier to their online responses. Correspondingly, only 6.5% of our respondents were from the ‘other’ education category, which included people with non-formal or no education.

Five QQ groups and three WeChat groups were randomly selected for the sample; however, there are thousands of QQ and WeChat groups across China, which were missed. Therefore, there was a likelihood of non-response bias, which was tested using the poplar approach of comparing the early and late responses [26, 90, 91], through which the late respondents (who took longer to respond) were believed to have the same responses (characteristics) as the non-respondents [92]. Here, in contrast to conventional mail (email) surveys, no invitations were sent to specific respondents for the voluntary survey; instead, QR codes were posted in WeChat and QQ groups, and most of the responses were received on the first day of the posting. Therefore, the time taken to answer the questionnaire is considered as an indicator of late responses, where the respondents who take longer than the average time of 110 seconds to completely answer the questions are considered to be late respondents (early: n = 102, 65.8% and late: n = 53, 34.2%). The average of the responses from the early and late response groups was taken across different questions to perform the independent sample t-test in SPSS (version 23). In the first step, the assumption of homogeneity of variances was tested and satisfied, using the ‘Levene test for equality of variances’, in which the null hypothesis of homogeneity (equality) of variance across samples was accepted at p = 0.56. The independent t-test (2-tailed) was rejected at p = 0.65 (95% CI = -0.54, 0.34), indicating that there was no statistically significant difference between the means of the responses (averaged) between the early and the late groups. Therefore, the t-test suggested that there was no problem of non-response bias with the sample of this study.

The convenience sampling approach was preferred to the more reliable conventional approaches (e.g. face-to-face, telephonic, and postal surveys). The current sample size of 155 respondents was determined using recent green supply chain/ logistics literature, which stated that a sample size of 100–200 observations was adequate for this type of research problem [26]. Furthermore, for survey research, generally, a minimum sample size of N = 100 is suggested [49]; thus our sample size of N = 155 valid respondents exceeded the minimum criterion. The sample error was ±8% at a 95% confidence level for the study’s sample size of 155. Therefore, future studies could consider a larger sample size to minimise sample errors. Additionally, the respondents’ demographics could be investigated in relation to the Chinese consumers’ willingness to participate in CEP companies’ green logistics.

## 6. Conclusions

Logistics operations, particularly CEP logistics, have a significant environmental impact. China is the global leader in the CEP industry. In recent years, the Chinese CEP industry has seen an average annual increase of 35% in the number of parcels, while the industry is expected to achieve an 8% ‘compound annual growth rate (CAGR)’ in the coming years. This rapid growth of the Chinese CEP industry will further increase the Chinese CEP logistics’ environmental footprint. The solution is to adopt green CEP logistics, which requires consumer participation. Consumer participation in the Chinese green product market, however, is very low, while the green market lacks competitiveness. In particular, Chinese consumers’ willingness to participate in CEP companies’ green logistics is not well known. This study filled this research gap. Additionally, the study addressed some technical issues with related survey-based literature. The data were collected from 155 demographically diversified final respondents. A total of 15 items, with three latent factors (economic, operational, and social) were identified. All the items and factors had a significant (p < 0.001, N = 155) positive correlation with consumer willingness. Furthermore, consumers were highly likely to not participate in direct and indirect economic activities. They were, however, more likely to participate in operational and social factors. Therefore, it is recommended that the government should provide monetary incentives to CEP companies that adopt green logistics, such as tax reductions and subsidies, to reduce the cost of adopting green logistics. Meanwhile, the CEP industry should provide some direct and indirect incentives for reusing, recycling, and sharing materials, and spending time on awareness of green logistics to improve the likelihood of consumers’ participation in economic factors. The government and CEP industry can further improve the operational and social factors’ effects on consumer willingness through advertising, environmental education at schools and colleges, community help centres, and other non-monetary incentives, such as green citizen badges, seminars, and short courses and certifications. Future research with a larger sample, with participants from more diversified backgrounds, including from different nationalities and ethnicities, could shed more light on this topic.

## Supporting information

S1 Table Sample of the questionnaire in English. Where the options include strongly negative, negative, neutral, positive and strongly positive.
(DOCX)

S2 Table Sample of the questionnaire in Chinese. Where the options include strongly negative, negative, neutral, positive and strongly positive.
(DOCX)

S3 Table Answers to the open ended optional question. “Suggestions for active participation of customers in green logistics of CEP.”
(DOCX)

S4 Table Items and factor loadings (covariances), standard errors, and z-test scores for Confirmatory Factor Analysis (CFA). (DOCX)

S1 Appendix Notes on the appropriateness and execution of Exploratory Factor Analysis (EFA) with Principal axis factor extraction (PFA). (DOCX)

S2 Appendix A note on the statistical significance and confidence intervals associated with multinomial logistic regression. (DOCX)

S3 Appendix Equations pertaining to the study’s results section. (DOCX)

S1 File This file contains data on the dependent and independent variables. The data is collected via an online survey of 155 respondents. The latent factor scores (values) are calculated by averaging the values of variables belonging to the same factor.
(XLSX)
