# Supplementary material for: A methodologically sound survey of Chinese consumers’ willingness to participate in courier, express, and parcel companies’ green logistics
Source: PLoS One. 2021 Jul 30;16(7):e0255532. doi: 10.1371/journal.pone.0255532 (PMC8323873; doi:10.1371/journal.pone.0255532)
Supplement: S1 Table — Where the options include strongly negative, negative, neutral, positive and strongly positive. (DOCX) [file pone.0255532.s001.docx]

**S1 Table. Sample of the English questionnaire.** Where the options include strongly negative, negative, neutral, positive and strongly positive.

| **Title: A methodologically sound survey of Chinese consumers' willingness to participate in courier, express, and parcel (CEP) companies' green logistics** | | | | | |
| --- | --- | --- | --- | --- | --- |
| Dear participants, thank you very much for taking the time to fill out this survey. The purpose of this study is to develop an understanding of the factors affecting Chinese consumers' willingness to participate in express companies' green logistics. Please answer the following questions based on your current situation. There are no correct or incorrect answers. The questionnaire will be completed anonymously. Your response will have no negative consequences. The survey results are only used for research statistics. Please click on the appropriate option. Participants under the age of 18 (minors) must submit a signed parental/guardian consent form, which must be scanned and sent to the corresponding author via WeChat, QQ, or email. Thank you once more for your help and participation. | | | | | |
| 1. What is your gender? 2. Male 3. Female 4. What is the highest degree or level of education you have completed? 5. Bachelor degree 6. Master degree or higher 7. Middle school 8. Primary school 9. Technical secondary school 10. Other 11. Where do you currently live? 12. Village 13. City centre 14. Town 15. Suburb 16. Which of the following categories best describes your employment status? 17. Full-time employment 18. Part-time employment 19. Full-time student 20. Part-time student 21. Unpaid (e.g. Volunteer) 22. Dependent (e.g. Children, Old age) 23. Housewife 24. Other 25. Which of the following describes your annual income? 26. 0 27. 1 – 9,999 RMB 28. 10000 – 24,999 RMB 29. 25,000 – 49,999 RMB 30. 50,000 –74,999 RMB 31. 75,000 – 99,999 RMB 32. 100,000 – 149,999 RMB 33. More than 150,000 RMB | | | | | |
| Items | Strongly negative | Negative | Neutral | Positive | Strongly Positive |
| 1. I am willing to pay for green logistics (Payment)? |  |  |  |  |  |
| 1. I support CEP companies to develop green logistics (Support)? |  |  |  |  |  |
| 1. I am willing to spend time understanding the green logistics of CEP (Time spent)? |  |  |  |  |  |
| 1. I will consider environmental issues in my daily life (Environmental consideration)? |  |  |  |  |  |
| 1. I will take the old parcel bag for reuse to the CEP delivery office (Reuse)? |  |  |  |  |  |
| 1. I am willing to use a shared parcel box (Shared boxes)? |  |  |  |  |  |
| 1. If the express box contains instructions for recycling, I'll take the time to discard the package as directed (Recycling)? |  |  |  |  |  |
| 1. I agree that everyone should respond positively to the development of green logistics (Positive response)? |  |  |  |  |  |
| 1. I would like to pick up the package at a public (shared) delivery point instead of directly at home (Shared pickup locations)? |  |  |  |  |  |
| 1. In order to develop green logistics, I would like to see express delivery companies increase their fees (Raising fee)? |  |  |  |  |  |
| 1. The community where I live helps me to understand the green logistics of CEP companies (Community help)? |  |  |  |  |  |
| 1. I agree that CEP companies should include green logistics in their corporate strategy (Corporate strategy)? |  |  |  |  |  |
| 1. I would like to see reduced material, inner cushions, tape, etc. used on parcel packaging by CEP companies (Ecological working)? |  |  |  |  |  |
| 1. I am willing to be a publicity volunteer for the development of green logistics (Volunteering)? |  |  |  |  |  |
| 1. My willingness to CEP’s green logistics is (Dependent variable)? |  |  |  |  |  |
| 1. How can consumers actively participate in the green logistics of CEP (Optional)? |  | | | | |
