# Supplementary material for: A methodologically sound survey of Chinese consumers’ willingness to participate in courier, express, and parcel companies’ green logistics
Source: PLoS One. 2021 Jul 30;16(7):e0255532. doi: 10.1371/journal.pone.0255532 (PMC8323873; doi:10.1371/journal.pone.0255532)
Supplement: S2 Table — Where the options include strongly negative, negative, neutral, positive and strongly positive. (DOCX) [file pone.0255532.s002.docx]

**S2 Table. Sample of the Chinese questionnaire.** Where the options include strongly negative, negative, neutral, positive and strongly positive.

| **Title: A methodologically sound survey of Chinese consumers' willingness to participate in courier, express, and parcel (CEP) companies' green logistics** | | | | | |
| --- | --- | --- | --- | --- | --- |
| 亲爱的参与者，非常感谢您抽出宝贵时间填写此调查表。这项研究的目的是加深对影响中国消费者参与快递公司绿色物流意愿因素的理解。请根据您的当前情况回答以下问题。没有所谓正确或错误的答案。问卷将以匿名方式填写。您的回答不会有负面影响。调查结果仅用于研究统计。请单击适当的选项。未满18岁（未成年）的参与者必须提交父母/监护人签署的同意书，必须将其扫描并通过微信，QQ或电子邮件发送给相应的作者。再次感谢您的帮助和参与。 | | | | | |
| 1. 你的性别是什么?   1. 男性 2. 女性   2. 你所完成的最高学历或教育程度是什么?   1. 本科 2. 硕士及以上学历 3. 中学 4. 小学 5. 中专 6. 其他   3. 你目前住在哪里?   1. 农村 2. 市中心 3. 城镇 4. 郊区   4. 以下哪一项最能描述你的就业状况?   1. 全职工作 2. 兼职工作 3. 全日制学生 4. 兼职学生 5. 无偿(如志愿者) 6. 依赖(如儿童、老年) 7. 家庭主妇 8. 其他   5. 以下哪项描述了你的年收入?   1. 0 2. 1 — 9,999元 3. 10,000 — 24,999元 4. 25,000 — 49,999元 5. 50,000 — 74,999元 6. 75,000 — 99,999元 7. 100,000 — 149,999元 8. 150,000元以上 | | | | | |
| 问题 | 非常消极 | 消极 | 中立 | 积极 | 非常积极 |
| 我愿意为绿色物流买单? |  |  |  |  |  |
| 我支持快递企业发展绿色物流? |  |  |  |  |  |
| 我愿意花时间了解快递企业绿色物流的运作? |  |  |  |  |  |
| 在我的日常生活中，我会考虑环保问题? |  |  |  |  |  |
| 我会把旧的购物袋带到商店再次使用? |  |  |  |  |  |
| 我愿意使用共享包装盒? |  |  |  |  |  |
| 如果在快递包装上写有可循环使用的拆装步骤，我会花时间按步骤拆件? |  |  |  |  |  |
| 我同意每个人都应积极响应绿色物流的发展? |  |  |  |  |  |
| 我愿意到公共(共享)的收派地点取包裹，而不是直接在家里收包裹? |  |  |  |  |  |
| 我愿意快递企业为了发展绿色物流而提高收费标准? |  |  |  |  |  |
| 我生活的社区有助于我了解快递企业的绿色物流? |  |  |  |  |  |
| 我同意快递企业应该把绿色物流纳入企业战略里? |  |  |  |  |  |
| 我愿意快递企业实行减量化包装，减重快递用包装纸箱、减少内缓冲物、开发免胶带纸箱、胶带宽度变窄等? |  |  |  |  |  |
| 我愿意做绿色物流发展的宣传志愿者? |  |  |  |  |  |
| 我的绿色购买意向是? |  |  |  |  |  |
| 让消费者积极参与快递企业绿色物流运作的方法有? |  | | | | |
