# Supplementary material for: A methodologically sound survey of Chinese consumers’ willingness to participate in courier, express, and parcel companies’ green logistics
Source: PLoS One. 2021 Jul 30;16(7):e0255532. doi: 10.1371/journal.pone.0255532 (PMC8323873; doi:10.1371/journal.pone.0255532)
Supplement: S3 Table — “Suggestions for active participation of customers in green logistics of CEP.” (DOCX) [file pone.0255532.s003.docx]

**S3 Table. Answers to the open ended optional question: “Suggestions for active participation of customers in green logistics of CEP.”**

| Number | Answers |
| --- | --- |
| 1 | Carry out green logistics packages |
| 2 | Cash subsidies |
| 3 | Reduce the price of green logistics and increase the price of non-green logistics |
| 4 | Price and promotion |
| 5 | To ensure the safety of express can be simple packaging |
| 6 | Green consumption to give gifts reward |
| 7 | Publicize to customers when sending and receiving couriers |
| 8 | Green recyclable packaging |
| 9 | Many advocates |
| 10 | Propaganda |
| 11 | On the door |
| 12 | Change the express delivery method |
| 13 | The state introduces corresponding policies |
| 14 | There is a certain reward system |
| 15 | Give some benefits |
| 16 | Transparent. The handling process is the basis of green logistics. The level of consumer participation depends on the service provider's attentiveness. The transparency and rationality of green logistics operation is an important guarantee for participation. |
