# Supplementary material for: A methodologically sound survey of Chinese consumers’ willingness to participate in courier, express, and parcel companies’ green logistics
Source: PLoS One. 2021 Jul 30;16(7):e0255532. doi: 10.1371/journal.pone.0255532 (PMC8323873; doi:10.1371/journal.pone.0255532)
Supplement: S4 Table — (DOCX) [file pone.0255532.s004.docx]

**S4 Table. Model factor loadings, standard errors and z-test scores.**

| **Items** | **Estimate** | **Std. Error** | **z-value** | **P(>\|z\|)** |
| --- | --- | --- | --- | --- |
| **Between latent factors and observed variables** |  |  |  |  |
| **Economic factor** |  |  |  |  |
| **Payment** | 1.000 | – | – | – |
| **Time spent** | 1.052 | 0.059 | 17.952 | < 0.001 |
| **Environmental consideration** | 1.098 | 0.057 | 19.253 | < 0.001 |
| **Reuse** | 0.861 | 0.063 | 13.577 | < 0.001 |
| **Shared boxes** | 0.841 | 0.067 | 12.471 | < 0.001 |
| **Recycling** | 1.003 | 0.062 | 16.269 | <0.001 |
| **Positive response** | 0.994 | 0.066 | 15.139 | < 0.001 |
| **Raising fee** | 0.968 | 0.060 | 16.188 | < 0.001 |
| **Operational factor** |  |  |  |  |
| **Support** | 1.000 | – | – | – |
| **Corporate strategy** | 0.876 | 0.061 | 14.406 | < 0.001 |
| **Ecological working** | 0.916 | 0.060 | 15.191 | < 0.001 |
| ***Social factor*** |  |  |  |  |
| **Shared pickup locations** | 1.000 | – | – | – |
| **Community help** | 0.896 | 0.080 | 11.270 | < 0.001 |
| **Volunteering** | 0.952 | 0.066 | 14.379 | < 0.001 |
| **Between latent factors** |  |  |  |  |
| **Economic–Operational** | 0.535 | 0.045 | 11.803 | < 0.001 |
| **Economic–Social** | 0.543 | 0.045 | 12.195 | < 0.001 |
| **Operational–Social** | 0.417 | 0.050 | 8.284 | < 0.001 |

* The z-test is a hypothesis test to determine if a single sample mean is significantly different from population mean [65]. The null hypothesis is rejected at P < 0.001. Note the critical value at P < 0.001 is 3.719 which is significantly less than the z-values of our model.
