# Supplementary material for: A methodologically sound survey of Chinese consumers’ willingness to participate in courier, express, and parcel companies’ green logistics
Source: PLoS One. 2021 Jul 30;16(7):e0255532. doi: 10.1371/journal.pone.0255532 (PMC8323873; doi:10.1371/journal.pone.0255532)
Supplement: S1 Appendix — (DOCX) [file pone.0255532.s005.docx]

### **Notes on the appropriateness and execution of Exploratory factor analysis (EFA) with Principal axis factor extraction (PFA)**

The Exploratory Factor Analysis (EFA) is used to define complex inter-relationships between items and group items that are part of the unified definitions [1]. The first stage of any EFA is to extract the factors during which the computer programme (like SPSS) explores the covariance between variables in an attempt to classify the factors underlying the data [2]. The most common extraction methods are the Principal Component Analysis (PCA) and the Principal (axis) Factor Analysis (PFA) [3]. In a strict sense, PCA is not a factor analysis, but the results are similar to those of the factor analysis [3]. Various researchers have strongly criticized the use of PCA as an EFA method [4, 5]. There are some statistical differences between the EFA and the PCA [2, 6]. This study uses the PFA extraction method for the purpose of the EFA analysis. According to the rule of thumb (Kaiser rule) an eigen value of >1 is selected for this study, which means that each factor will describe more variance than a single observed variable. Following this criterion, three factors presenting the economic, operational and social willingness of Chinese consumers to participate in the green logistics of express companies were generated by SPSS (version 23). These three factors explain a total variance of 58.24%. Where the first, second and third factors account for 44.55%, 8.38% and 5.31% of the total variance in all variables. For enhanced understanding, factors are rotated, as the non-rotated factors are unclear [7]. Two primary forms of rotation, orthogonal and oblique rotation, are available [8]. Orthogonal rotation, as factors are usually correlated to some degree, is less realistic [7]. *Direct Oblimin* or *Promax* are the most common types of oblique rotation [7, 8]. *Direct Oblimin* focuses on simplifying the structure and output statistics, while *Promax* is faster and therefore useful for larger datasets [7]. This study uses *Direct Oblimin* because the results (factor loadings) produced by this rotation method are easier to interpret. The oblique rotation generates two additional factor matrices called pattern and structure, and the pattern matrix should be used for analysis [8].

**References**

1. Polit DF, Beck CT. Nursing Research: Generating and Assessing Evidence for Nursing Practice. 9th ed. Philadelphia, USA: Wolters Klower Health, Lippincott Williams & Wilkins; 2012.

2. Plucker JA. Exploratory and Confirmatory Factor Analysis in Gifted Education : Examples With Self-Concept Data. J Educ Gift. 2003;27(1):20–35.

3. Beaumont R. An introduction to Principal Component Analysis & Factor Analysis Using SPSS 19 and R (psych package) [Internet]. Medical statistics made understandable. 2012 [cited 2021 Feb 6]. p. 24. Available from: http://www.floppybunny.org/robin/web/virtualclassroom/stats/statistics2/pca1.pdf

4. Pedhazur EJ, Schmelkin LP. Measurement, design, and analysis: An integrated approach. Hillsdale, NJ: Erlbaum; 1991.

5. Fabrigar LR, Wegener DT, Maccallum RC, Strahan EJ. Evaluating the Use of Exploratory Factor Analysis in Psychological Research. Psychol Methods. 1999;4(3):272–99.

6. Suhr DD. Statistics and Data Analysis Principal Component Analysis vs . Exploratory Factor Analysis. In: SUGI 30 [Internet]. Pennsylvania, USA; 2005. p. 1–11. Available from: https://support.sas.com/resources/papers/proceedings/proceedings/sugi30/203-30.pdf

7. Yong AG, Pearce S. A Beginner’s Guide to Factor Analysis : Focusing on Exploratory Factor Analysis. Tutor Quant Methods Psychol. 2013;9(2):79–94.

8. Samuels P. Advice on Exploratory Factor Analysis [Internet]. Birmingham, England.; 2017. Available from: http://www.open-access.bcu.ac.uk/id/eprint/6076
