# Supplementary material for: A methodologically sound survey of Chinese consumers’ willingness to participate in courier, express, and parcel companies’ green logistics
Source: PLoS One. 2021 Jul 30;16(7):e0255532. doi: 10.1371/journal.pone.0255532 (PMC8323873; doi:10.1371/journal.pone.0255532)
Supplement: S2 Appendix — (DOCX) [file pone.0255532.s006.docx]

# A note on the statistical significance and confidence intervals associated with multinomial logistic regression

Following satisfactory results on the significance of the complete model and its Pseudo R-Square, the significance for each item is not considered separately. The widespread use in social science of the .05 and .01 levels of significance is a question of a tradition with no scientific or rational basis [1]. To determine the scientific or practical significance of a set of observations, statistical significance is neither necessary nor appropriate [2]. In particular, in non-experimental research, such as the social sciences, tests of statistical significance in design and analysis, in theory and in practise, are not relevant [3].

The issue with confidence intervals is that they are calculated on the basis of the data's statistical significance. If we wish to avoid significance testing, we should avoid techniques such as confidence intervals, which are essentially inversions of significance tests [4]. Additionally, Richard et al. [5] demonstrate confidence fallacies via the widely recommended and frequently used confidence interval (CI) for ANOVA and regression analysis. They argue that invoking CI theory is superfluous in the best-case scenario, where inferences can be explained independently of it, and unreasonable in the worst-case scenario, where they cannot. As a result of the scepticism expressed by several statisticians and researchers regarding statistical significance (P) and confidence intervals, particularly in the context of regression analysis, we did not discard the results obtained through our MLR analysis despite the fact that some of the variables were non-significant. The most compelling argument against the non-significance of some of our individual variable MLR results is that these sample results should be interpreted cautiously when applied to the entire population. The MLR analysis results are shown in Table A.1.

**Table A.1. Results of the multinomial regression analysis (MLR).**

| **Group** | **Items** | **B** | **Std. Error** | **Wald** | **Sig.** | **Exp(B)** | **95% Confidence Interval for Exp(B)** | |
| --- | --- | --- | --- | --- | --- | --- | --- | --- |
|  |  |  |  |  |  |  | **Lower Bound** | **Upper Bound** |
| **Low willingness=2** | **Intercept** | -10.69 | 27.80 | 0.15 | 0.70 |  |  |  |
|  | **[Payment=1.00]** | 1.55 | 14.66 | 0.01 | 0.92 | 4.71 | 1.58E-12 | 1.40497E+13 |
|  | **[Payment=2.00]** | 1.87 | 15.39 | 0.02 | 0.90 | 6.48 | 5.11E-13 | 8.20213E+13 |
|  | **[Payment=3.00]** | 1.24 | 12.44 | 0.01 | 0.92 | 3.45 | 8.86E-11 | 1.34007E+11 |
|  | **[Payment=4.00]** | 0.80 | 10.08 | 0.01 | 0.94 | 2.22 | 5.78E-09 | 849199273.7 |
|  | **[Support=2.00]** | 0.13 | 44.35 | 0.00 | 1.00 | 1.13 | 2.01E-38 | 6.40E+37 |
|  | **[Support=3.00]** | -0.19 | 12.19 | 0.00 | 0.99 | 0.83 | 3.50E-11 | 19622828851 |
|  | **[Support=4.00]** | 0.55 | 7.60 | 0.01 | 0.94 | 1.73 | 5.87E-07 | 5075480.003 |
|  | **[Time spent=1.00]** | 2.16 | 31.12 | 0.01 | 0.95 | 8.65 | 2.78E-26 | 2.69016E+27 |
|  | **[Time spent=2.00]** | 2.36 | 20.56 | 0.01 | 0.91 | 10.61 | 3.34E-17 | 3.36765E+18 |
|  | **[Time spent=3.00]** | 2.26 | 13.17 | 0.03 | 0.86 | 9.58 | 5.86E-11 | 1.56598E+12 |
|  | **[Time spent=4.00]** | 2.19 | 11.39 | 0.04 | 0.85 | 8.92 | 1.80E-09 | 44122144827 |
|  | **[Environmental consideration=1.00]** | 2.73 | 52.26 | 0.00 | 0.96 | 15.37 | 5.01E-44 | 4.71E+45 |
|  | **[Environmental consideration=3.00]** | -0.02 | 13.73 | 0.00 | 1.00 | 0.98 | 2.01E-12 | 4.76798E+11 |
|  | **[Environmental consideration=4.00]** | -0.26 | 10.50 | 0.00 | 0.98 | 0.77 | 8.91E-10 | 661981750.5 |
|  | **[Reuse=2.00]** | -1.01 | 17.66 | 0.00 | 0.95 | 0.36 | 3.36E-16 | 3.92011E+14 |
|  | **[Reuse=3.00]** | 0.86 | 10.67 | 0.01 | 0.94 | 2.37 | 1.97E-09 | 2838661760 |
|  | **[Reuse=4.00]** | 0.51 | 8.96 | 0.00 | 0.96 | 1.66 | 3.89E-08 | 70716292.58 |
|  | **[Shared boxes=1.00]** | 0.09 | 35.63 | 0.00 | 1.00 | 1.10 | 5.13E-31 | 2.35534E+30 |
|  | **[Shared boxes=2.00]** | 0.40 | 16.85 | 0.00 | 0.98 | 1.49 | 6.71E-15 | 3.29473E+14 |
|  | **[Shared boxes=3.00]** | -0.27 | 13.44 | 0.00 | 0.98 | 0.76 | 2.78E-12 | 2.08882E+11 |
|  | **[Shared boxes=4.00]** | -0.34 | 13.09 | 0.00 | 0.98 | 0.71 | 5.15E-12 | 97740590352 |
|  | **[Recycling=1.00]** | 1.42 | 36.99 | 0.00 | 0.97 | 4.15 | 1.36E-31 | 1.26675E+32 |
|  | **[Recycling=2.00]** | 0.78 | 50.09 | 0.00 | 0.99 | 2.17 | 5.01E-43 | 9.40E+42 |
|  | **[Recycling=3.00]** | 0.02 | 13.58 | 0.00 | 1.00 | 1.02 | 2.82E-12 | 3.70617E+11 |
|  | **[Recycling=4.00]** | 0.34 | 11.65 | 0.00 | 0.98 | 1.40 | 1.71E-10 | 11561125166 |
|  | **[Positive response=3.00]** | 0.26 | 10.63 | 0.00 | 0.98 | 1.30 | 1.17E-09 | 1437550382 |
|  | **[Positive response=4.00]** | 0.60 | 7.36 | 0.01 | 0.94 | 1.82 | 9.84E-07 | 3350098.951 |
|  | **[Shared pickups locations=1.00]** | -2.12 | 29.91 | 0.01 | 0.94 | 0.12 | 4.20E-27 | 3.45457E+24 |
|  | **[Shared pickups locations=2.00]** | 0.80 | 16.47 | 0.00 | 0.96 | 2.23 | 2.15E-14 | 2.32338E+14 |
|  | **[Shared pickups locations=3.00]** | -0.01 | 12.32 | 0.00 | 1.00 | 0.99 | 3.21E-11 | 30277568830 |
|  | **[Shared pickups locations=4.00]** | -0.59 | 11.28 | 0.00 | 0.96 | 0.56 | 1.40E-10 | 2196104247 |
|  | **[Raising fee=1.00]** | -1.28 | 28.30 | 0.00 | 0.96 | 0.28 | 2.28E-25 | 3.37469E+23 |
|  | **[Raising fee=2.00]** | -1.47 | 26.75 | 0.00 | 0.96 | 0.23 | 3.89E-24 | 1.36302E+22 |
|  | **[Raising fee=3.00]** | -0.41 | 26.48 | 0.00 | 0.99 | 0.67 | 1.94E-23 | 2.28281E+22 |
|  | **[Raising fee=4.00]** | -0.94 | 26.91 | 0.00 | 0.97 | 0.39 | 4.89E-24 | 3.14534E+22 |
|  | **[Community help=1.00]** | 1.55 | 36.48 | 0.00 | 0.97 | 4.72 | 4.17E-31 | 5.34376E+31 |
|  | **[Community help=2.00]** | 1.92 | 33.81 | 0.00 | 0.96 | 6.79 | 1.13E-28 | 4.09182E+29 |
|  | **[Community help=3.00]** | 1.59 | 33.77 | 0.00 | 0.96 | 4.89 | 8.74E-29 | 2.74044E+29 |
|  | **[Community help=4.00]** | 2.12 | 34.74 | 0.00 | 0.95 | 8.35 | 2.23E-29 | 3.12116E+30 |
|  | **[Corporate strategy=2.00]** | 155.70 | 0.00 | . | . | 4.15E+67 | 4.15E+67 | 4.15E+67 |
|  | **[Corporate strategy=3.00]** | -0.98 | 12.38 | 0.01 | 0.94 | 0.38 | 1.09E-11 | 12960201096 |
|  | **[Corporate strategy=4.00]** | -0.81 | 7.85 | 0.01 | 0.92 | 0.44 | 9.20E-08 | 2138541.788 |
|  | **[Ecological work=3.00]** | 0.98 | 10.46 | 0.01 | 0.93 | 2.68 | 3.35E-09 | 2138088604 |
|  | **[Ecological work=4.00]** | 1.04 | 7.48 | 0.02 | 0.89 | 2.82 | 1.22E-06 | 6514368.884 |
|  | **[Volunteering=1.00]** | 1.93 | 22.39 | 0.01 | 0.93 | 6.88 | 6.08E-19 | 7.78543E+19 |
|  | **[Volunteering=2.00]** | 1.82 | 15.56 | 0.01 | 0.91 | 6.18 | 3.50E-13 | 1.0897E+14 |
|  | **[Volunteering=3.00]** | 2.47 | 12.37 | 0.04 | 0.84 | 11.83 | 3.49E-10 | 4.00375E+11 |
|  | **[Volunteering=4.00]** | 2.65 | 11.53 | 0.05 | 0.82 | 14.14 | 2.17E-09 | 92045327479 |
| **Moderate willingness=3** | **Intercept** | -6.52 | 3.75 | 3.03 | 0.08 |  |  |  |
|  | **[Payment=1.00]** | 1.87 | 2.36 | 0.63 | 0.43 | 6.47 | 0.064 | 656.711 |
|  | **[Payment=2.00]** | 1.39 | 2.64 | 0.28 | 0.60 | 4.00 | 0.022 | 710.306 |
|  | **[Payment=3.00]** | 0.87 | 2.08 | 0.18 | 0.68 | 2.39 | 0.04 | 141.046 |
|  | **[Payment=4.00]** | 0.33 | 1.58 | 0.04 | 0.84 | 1.39 | 0.062 | 30.792 |
|  | **[Support=2.00]** | -0.49 | 9.15 | 0.00 | 0.96 | 0.61 | 1.01E-08 | 37229132.46 |
|  | **[Support=3.00]** | -0.32 | 2.35 | 0.02 | 0.89 | 0.73 | 0.007 | 72.235 |
|  | **[Support=4.00]** | 0.66 | 1.36 | 0.23 | 0.63 | 1.93 | 0.135 | 27.482 |
|  | **[Time spent=1.00]** | 3.57 | 7.18 | 0.25 | 0.62 | 35.43 | 2.77E-05 | 45384205.19 |
|  | **[Time spent=2.00]** | 4.77 | 4.11 | 1.35 | 0.25 | 117.77 | 0.037 | 372478.82 |
|  | **[Time spent=3.00]** | 3.18 | 2.23 | 2.03 | 0.15 | 24.12 | 0.303 | 1919.936 |
|  | **[Time spent=4.00]** | 2.87 | 1.91 | 2.25 | 0.13 | 17.54 | 0.417 | 738.2 |
|  | **[Environmental consideration=1.00]** | 4.85 | 10.80 | 0.20 | 0.65 | 128.28 | 8.24E-08 | 1.99658E+11 |
|  | **[Environmental consideration=3.00]** | 0.52 | 2.44 | 0.05 | 0.83 | 1.68 | 0.014 | 199.317 |
|  | **[Environmental consideration=4.00]** | -0.44 | 1.82 | 0.06 | 0.81 | 0.65 | 0.018 | 22.879 |
|  | **[Reuse=2.00]** | -0.40 | 3.37 | 0.01 | 0.91 | 0.67 | 0.001 | 498.194 |
|  | **[Reuse=3.00]** | 1.23 | 1.88 | 0.43 | 0.51 | 3.42 | 0.086 | 135.665 |
|  | **[Reuse=4.00]** | 0.59 | 1.53 | 0.15 | 0.70 | 1.80 | 0.089 | 36.392 |
|  | **[Shared boxes=1.00]** | -0.50 | 7.90 | 0.00 | 0.95 | 0.61 | 1.14E-07 | 3238580.907 |
|  | **[Shared boxes=2.00]** | 1.34 | 3.19 | 0.18 | 0.67 | 3.83 | 0.007 | 1983.983 |
|  | **[Shared boxes=3.00]** | 0.12 | 2.34 | 0.00 | 0.96 | 1.13 | 0.012 | 109.976 |
|  | **[Shared boxes=4.00]** | 0.05 | 2.16 | 0.00 | 0.98 | 1.05 | 0.015 | 72.823 |
|  | **[Recycling=1.00]** | 2.39 | 7.93 | 0.09 | 0.76 | 10.93 | 1.95E-06 | 61390298.95 |
|  | **[Recycling=2.00]** | -0.73 | 10.66 | 0.01 | 0.95 | 0.48 | 4.05E-10 | 570099809.5 |
|  | **[Recycling=3.00]** | -0.33 | 2.26 | 0.02 | 0.89 | 0.72 | 0.009 | 60.361 |
|  | **[Recycling=4.00]** | 0.33 | 1.90 | 0.03 | 0.86 | 1.39 | 0.033 | 58.122 |
|  | **[Positive response=3.00]** | 1.02 | 2.05 | 0.25 | 0.62 | 2.76 | 0.05 | 153.661 |
|  | **[Positive response=4.00]** | 0.96 | 1.22 | 0.62 | 0.43 | 2.61 | 0.241 | 28.324 |
|  | **[Shared pickups locations=1.00]** | -3.45 | 5.26 | 0.43 | 0.51 | 0.03 | 1.06E-06 | 951.095 |
|  | **[Shared pickups locations=2.00]** | -0.30 | 3.15 | 0.01 | 0.92 | 0.74 | 0.002 | 352.11 |
|  | **[Shared pickups locations=3.00]** | -0.27 | 2.06 | 0.02 | 0.90 | 0.76 | 0.013 | 43.036 |
|  | **[Shared pickups locations=4.00]** | -1.02 | 1.90 | 0.29 | 0.59 | 0.36 | 0.009 | 14.847 |
|  | **[Raising fee=1.00]** | -2.24 | 3.46 | 0.42 | 0.52 | 0.11 | 0 | 93.108 |
|  | **[Raising fee=2.00]** | -2.56 | 3.12 | 0.67 | 0.41 | 0.08 | 0 | 35.049 |
|  | **[Raising fee=3.00]** | -1.70 | 3.11 | 0.30 | 0.58 | 0.18 | 0 | 80.309 |
|  | **[Raising fee=4.00]** | -2.37 | 3.12 | 0.57 | 0.45 | 0.09 | 0 | 42.821 |
|  | **[Community help=1.00]** | 1.46 | 4.94 | 0.09 | 0.77 | 4.29 | 0 | 69263.3 |
|  | **[Community help=2.00]** | 1.92 | 4.17 | 0.21 | 0.65 | 6.83 | 0.002 | 24332.14 |
|  | **[Community help=3.00]** | 1.56 | 4.15 | 0.14 | 0.71 | 4.76 | 0.001 | 16111.571 |
|  | **[Community help=4.00]** | 2.45 | 4.35 | 0.32 | 0.57 | 11.61 | 0.002 | 58013.695 |
|  | **[Corporate strategy=2.00]** | -0.14 | 0.00 | . | . | 0.87 | 0.867 | 0.867 |
|  | **[Corporate strategy=3.00]** | -0.22 | 2.45 | 0.01 | 0.93 | 0.81 | 0.007 | 97.447 |
|  | **[Corporate strategy=4.00]** | -0.81 | 1.40 | 0.34 | 0.56 | 0.44 | 0.029 | 6.884 |
|  | **[Ecological work=3.00]** | 1.32 | 2.01 | 0.43 | 0.51 | 3.75 | 0.073 | 193.207 |
|  | **[Ecological work=4.00]** | 0.85 | 1.29 | 0.43 | 0.51 | 2.34 | 0.186 | 29.384 |
|  | **[Volunteering=1.00]** | 2.08 | 4.52 | 0.21 | 0.65 | 8.04 | 0.001 | 56703.438 |
|  | **[Volunteering=2.00]** | 3.12 | 2.41 | 1.67 | 0.20 | 22.69 | 0.201 | 2567.13 |
|  | **[Volunteering=3.00]** | 3.62 | 1.86 | 3.78 | 0.05 | 37.25 | 0.971 | 1429.588 |
|  | **[Volunteering=4.00]** | 3.26 | 1.75 | 3.44 | 0.06 | 25.91 | 0.832 | 806.747 |
| **High willingness=4** | **Intercept** | -6.59 | 3.38 | 3.81 | 0.05 |  |  |  |
|  | **[Payment=1.00]** | 1.77 | 1.90 | 0.87 | 0.35 | 5.87 | 0.143 | 240.849 |
|  | **[Payment=2.00]** | 2.66 | 2.23 | 1.42 | 0.23 | 14.34 | 0.18 | 1143.659 |
|  | **[Payment=3.00]** | 1.79 | 1.68 | 1.14 | 0.29 | 6.01 | 0.222 | 162.261 |
|  | **[Payment=4.00]** | 1.29 | 1.13 | 1.31 | 0.25 | 3.65 | 0.397 | 33.502 |
|  | **[Support=2.00]** | 0.53 | 9.85 | 0.00 | 0.96 | 1.71 | 7.01E-09 | 415612986 |
|  | **[Support=3.00]** | -0.16 | 2.27 | 0.01 | 0.94 | 0.85 | 0.01 | 73.087 |
|  | **[Support=4.00]** | 0.63 | 1.14 | 0.30 | 0.59 | 1.87 | 0.199 | 17.545 |
|  | **[Time spent=1.00]** | 1.88 | 7.02 | 0.07 | 0.79 | 6.54 | 6.89E-06 | 6209317.032 |
|  | **[Time spent=2.00]** | 1.53 | 3.93 | 0.15 | 0.70 | 4.61 | 0.002 | 10107.06 |
|  | **[Time spent=3.00]** | 2.30 | 1.75 | 1.73 | 0.19 | 10.01 | 0.322 | 311.054 |
|  | **[Time spent=4.00]** | 2.36 | 1.32 | 3.19 | 0.07 | 10.63 | 0.793 | 142.374 |
|  | **[Environmental consideration=1.00]** | 2.17 | 11.20 | 0.04 | 0.85 | 8.78 | 2.59E-09 | 29705843686 |
|  | **[Environmental consideration=3.00]** | -0.36 | 2.22 | 0.03 | 0.87 | 0.70 | 0.009 | 54.434 |
|  | **[Environmental consideration=4.00]** | -0.23 | 1.47 | 0.02 | 0.88 | 0.80 | 0.045 | 14.196 |
|  | **[Reuse=2.00]** | -1.66 | 3.24 | 0.26 | 0.61 | 0.19 | 0 | 109.288 |
|  | **[Reuse=3.00]** | 0.87 | 1.67 | 0.27 | 0.60 | 2.38 | 0.09 | 62.684 |
|  | **[Reuse=4.00]** | 0.59 | 1.25 | 0.22 | 0.64 | 1.81 | 0.155 | 21.069 |
|  | **[Shared boxes=1.00]** | 0.48 | 6.92 | 0.01 | 0.95 | 1.62 | 2.08E-06 | 1258186.454 |
|  | **[Shared boxes=2.00]** | -0.08 | 2.73 | 0.00 | 0.98 | 0.93 | 0.004 | 196.274 |
|  | **[Shared boxes=3.00]** | -0.58 | 1.78 | 0.11 | 0.74 | 0.56 | 0.017 | 18.422 |
|  | **[Shared boxes=4.00]** | -0.68 | 1.57 | 0.19 | 0.67 | 0.51 | 0.024 | 10.959 |
|  | **[Recycling=1.00]** | 1.21 | 8.07 | 0.02 | 0.88 | 3.36 | 4.51E-07 | 25070146.71 |
|  | **[Recycling=2.00]** | 1.90 | 11.29 | 0.03 | 0.87 | 6.70 | 1.63E-09 | 27476034516 |
|  | **[Recycling=3.00]** | 0.24 | 2.00 | 0.02 | 0.90 | 1.27 | 0.025 | 64.116 |
|  | **[Recycling=4.00]** | 0.44 | 1.50 | 0.08 | 0.77 | 1.55 | 0.082 | 29.337 |
|  | **[Positive response=3.00]** | -0.13 | 1.98 | 0.00 | 0.95 | 0.88 | 0.018 | 42.144 |
|  | **[Positive response=4.00]** | 0.54 | 0.98 | 0.30 | 0.58 | 1.71 | 0.252 | 11.562 |
|  | **[Shared pickups locations=1.00]** | -1.87 | 4.68 | 0.16 | 0.69 | 0.15 | 1.61E-05 | 1466.715 |
|  | **[Shared pickups locations=2.00]** | 1.69 | 2.93 | 0.33 | 0.56 | 5.44 | 0.017 | 1705.012 |
|  | **[Shared pickups locations=3.00]** | 0.14 | 1.86 | 0.01 | 0.94 | 1.15 | 0.03 | 44.031 |
|  | **[Shared pickups locations=4.00]** | -0.48 | 1.68 | 0.08 | 0.77 | 0.62 | 0.023 | 16.476 |
|  | **[Raising fee=1.00]** | -1.04 | 3.39 | 0.09 | 0.76 | 0.35 | 0 | 272.864 |
|  | **[Raising fee=2.00]** | -1.20 | 3.01 | 0.16 | 0.69 | 0.30 | 0.001 | 110.227 |
|  | **[Raising fee=3.00]** | 0.27 | 3.02 | 0.01 | 0.93 | 1.31 | 0.004 | 488.467 |
|  | **[Raising fee=4.00]** | -0.32 | 3.03 | 0.01 | 0.92 | 0.73 | 0.002 | 278.059 |
|  | **[Community help=1.00]** | 2.03 | 4.68 | 0.19 | 0.67 | 7.59 | 0.001 | 72493.833 |
|  | **[Community help=2.00]** | 2.43 | 3.90 | 0.39 | 0.53 | 11.30 | 0.005 | 23537.31 |
|  | **[Community help=3.00]** | 2.03 | 3.91 | 0.27 | 0.60 | 7.62 | 0.004 | 16293.201 |
|  | **[Community help=4.00]** | 2.49 | 4.04 | 0.38 | 0.54 | 12.07 | 0.004 | 32862.113 |
|  | **[Corporate strategy=2.00]** | -0.50 | 0.00 | . | . | 0.61 | 0.608 | 0.608 |
|  | **[Corporate strategy=3.00]** | -1.71 | 2.34 | 0.53 | 0.47 | 0.18 | 0.002 | 17.7 |
|  | **[Corporate strategy=4.00]** | -1.03 | 1.18 | 0.77 | 0.38 | 0.36 | 0.035 | 3.592 |
|  | **[Ecological work=3.00]** | 1.04 | 1.88 | 0.31 | 0.58 | 2.84 | 0.072 | 112.047 |
|  | **[Ecological work=4.00]** | 1.43 | 1.10 | 1.69 | 0.19 | 4.18 | 0.485 | 36.094 |
|  | **[Volunteering=1.00]** | 2.35 | 4.32 | 0.30 | 0.59 | 10.50 | 0.002 | 49953.82 |
|  | **[Volunteering=2.00]** | 1.52 | 2.30 | 0.44 | 0.51 | 4.55 | 0.051 | 409.867 |
|  | **[Volunteering=3.00]** | 2.43 | 1.53 | 2.53 | 0.11 | 11.40 | 0.567 | 229.119 |
|  | **[Volunteering=4.00]** | 2.99 | 1.40 | 4.60 | 0.03 | 19.89 | 1.293 | 306.067 |

*The reference category is: 5.00 for both the dependent and the independent variables.

# References

1. Skipper JK, Guenther AL, Nass G, The S, Sociologist A, Feb N. A Note concerning the Uses of Statistical Levels of Significance in Social Science. Am Sociol. 1967;2(1):16–8.

2. Greenland S, Senn SJ, Rothman KJ, Carlin JB, Poole C, Goodman SN, et al. Statistical tests , P values , confidence intervals , and power : a guide to misinterpretations. Eur J Epidemiol. 2016;31(4):337–50.

3. Selvin HC. A Critique of Tests of Significance in Survey Research. Am Sociol Rev. 1957;22(5):519–27.

4. Steiger JH, Fouladi RT. Noncentrality interval estimation and the evaluation of statistical models. In: Harlow L, Mulaik S, Steiger J, editors. What if there were no significance tests? [Internet]. Mahwah, New Jersey: Erlbaum.; 1997. p. 221–257. Available from: https://www.routledge.com/What-If-There-Were-No-Significance-Tests-Classic-Edition/Harlow-Mulaik-Steiger/p/book/9781138892477

5. Morey RD, Hoekstra R, Rouder JN, Lee MD, Wagenmakers E. The fallacy of placing confidence in confidence intervals. Psychon Bull Rev. 2016;23:103–23.
