# Supplementary material for: A methodologically sound survey of Chinese consumers’ willingness to participate in courier, express, and parcel companies’ green logistics
Source: PLoS One. 2021 Jul 30;16(7):e0255532. doi: 10.1371/journal.pone.0255532 (PMC8323873; doi:10.1371/journal.pone.0255532)
Supplement: S3 Appendix — (DOCX) [file pone.0255532.s007.docx]

This appendix section contains the mathematical equations underlying the primary models used to derive the study's results. Software programs, such as the SPSS program (version 23) used in this study, can assist users in automatically applying models and deriving results.

# **Descriptive statistics**

## **Arithmetic mean**

The formula for calculating the athematic mean or average is given below.

$$\begin{aligned} \bar{V}=\frac{\sum_{m=1}^{q} V_{i}}{Q}\#\left( A.1 \right) \end{aligned}$$

Where $\bar{V}$ represents the variable of interest's arithmetic mean. $V_{i}$ represents the value of the $mth$ observation, and $Q$ represents the total number of observations.

## **Sample standard deviation**

The sample standard deviation is the default measure of standard deviation in the SPSS software [1].

$$\begin{aligned} sV=\sqrt{\frac{\sum_{m=1}^{q} V_{i}-\bar{V}}{Q-1}}\#\left( A.2 \right) \end{aligned}$$

Where $s$ represents the sample standard deviation for the variable $V$.

## **Range**

The range of a set of observations or responses to a given variable is estimated by subtracting the maximum and minimum values associated with the set of observations.

$$\begin{aligned} RV=V_{max}-V_{min}\#\left( A.3 \right) \end{aligned}$$

Where $RV$ represents a variable's range value. $V_{max}$ displays the maximum value of a variable, while $V_{min}$ displays the minimum value.

# **Correlation analysis**

## **Pearson correlation**

The bivariate Pearson correlation, which represents the directional relationship between the dependent and independent variables, can be calculated using the following equation.

$$\begin{aligned} r_{D,T}=\frac{\sum_{m=1}^{q} \left( D_{m}-\bar{D} \right)\left( T_{m}-\bar{T} \right)}{\sqrt{\sum_{m=1}^{q} \left( D_{m}-\bar{D} \right)^{2}}\sqrt{\sum_{m=1}^{q} \left( T_{m}-\bar{T} \right)^{2}}}\#\left( A.4 \right) \end{aligned}$$

Where $r_{D,T}$ represents the Pearson correlation between the dependent variable $D$ and the independent variable $T (T=1,2,3,\cdots,n)$. And $m (m=1,2,3,\cdots,q)$ represents the responses received for a specific question.

# **Multinomial logistic regression analysis**

The following equation represents the maximum odds ratio for our model with willingness as the outcome variable.

$$\begin{aligned} ln\left( \frac{O\left( Willingness=W_{i-r} \right)}{O\left( Willingness=W_{r} \right)} \right)=b_{i0}+b_{i1}+b_{i2}+\cdots+b_{in}\#\left( A.5 \right) \end{aligned}$$

Where $ln\left( \frac{O\left( Willingness=W_{i-r} \right)}{O\left( Willingness=W_{r} \right)} \right)$ represents the log odds of being in one group versus the reference group when moving between independent variable score levels [2]. $W_{i-r}$ presents all willingness groups except for the reference group r. The reference group is presented by $W_{r}$. We can obtain the value of Exp (B) by multiplying the exponent by the log odds (B) [3].

$$\begin{aligned} Exp\left( B \right)=Exp\left[ ln\left( \frac{O\left( Willingness=W_{i-r} \right)}{O\left( Willingness=W_{r} \right)} \right) \right]\#\left( A.6 \right) \end{aligned}$$

# **References**

1. Berg RG van den. Standard Deviation – What Is It? [Internet]. Statistics A-Z. [cited 2021 Apr 5]. Available from: https://www.spss-tutorials.com/standard-deviation/

2. UCLA. MULTINOMIAL LOGISTIC REGRESSION | SPSS DATA ANALYSIS EXAMPLES [Internet]. Statistical Consulting Group. [cited 2021 Apr 4]. Available from: https://stats.idre.ucla.edu/spss/dae/multinomial-logistic-regression/

3. Strand S, Cadwallader S, Firth D. 4.2 An Introduction to Odds, Odds Ratios and Exponents. In: Using Statistical Regression Methods in Education Research [Internet]. University of Southampton; 2011. p. 2. Available from: https://www.restore.ac.uk/srme/www/fac/soc/wie/research-new/srme/modules/mod4/2/index.html
